# The *WWOX/HIF1A* Axis Downregulation Alters Glucose Metabolism and Predispose to Metabolic Disorders

**DOI:** 10.3390/ijms23063326

**Published:** 2022-03-19

**Authors:** Izabela Baryła, Ewa Styczeń-Binkowska, Elżbieta Płuciennik, Katarzyna Kośla, Andrzej K. Bednarek

**Affiliations:** Department of Molecular Carcinogenesis, Medical University of Lodz, 90-752 Lodz, Poland; ewa.styczen-Binkowska@umed.lodz.pl (E.S.-B.); elzbieta.pluciennik@umed.lodz.pl (E.P.); katarzyna.kosla@umed.lodz.pl (K.K.); andrzej.bednarek@umed.lodz.pl (A.K.B.)

**Keywords:** glycolysis, hypoxia-inducible factor 1α (HIF1α), WW domain-containing oxidoreductase (WWOX), metabolic disorders

## Abstract

Recent reports indicate that the hypoxia-induced factor (HIF1α) and the Warburg effect play an initiating role in glucotoxicity, which underlies disorders in metabolic diseases. WWOX has been identified as a HIF1α regulator. *WWOX* downregulation leads to an increased expression of HIF1α target genes encoding glucose transporters and glycolysis’ enzymes. It has been proven in the normoglycemic mice cells and in gestational diabetes patients. The aim of the study was to determine WWOX’s role in glucose metabolism regulation in hyperglycemia and hypoxia to confirm its importance in the development of metabolic disorders. For this purpose, the *WWOX* gene was silenced in human normal fibroblasts, and then cells were cultured under different sugar and oxygen levels. Thereafter, it was investigated how *WWOX* silencing alters the genes and proteins expression profile of glucose transporters and glycolysis pathway enzymes, and their activity. In normoxia normoglycemia, higher glycolysis genes expression, their activity, and the lactate concentration were observed in *WWOX* KO fibroblasts in comparison to control cells. In normoxia hyperglycemia, it was observed a decrease of insulin-dependent glucose uptake and a further increase of lactate. It likely intensifies hyperglycemia condition, which deepen the glucose toxic effect. Then, in hypoxia hyperglycemia, *WWOX* KO caused weaker glucose uptake and elevated lactate production. In conclusion, the *WWOX/HIF1A* axis downregulation alters glucose metabolism and probably predispose to metabolic disorders.

## 1. Introduction

Hyperglycemia and hypoxia are suggested to be important factors in the pathophysiology of metabolic disorders and their complication [1,2,3]. Hyperglycemia could cause cellular hypoxia. The obesity associated with simple sugars consumption also triggers hypoxia. Then, hypoxia activates and stabilizes hypoxia-inducible factor HIF1α signaling, resulting in adverse metabolic effects, including insulin resistance. Thus it is assumed that hypoxia-inducible factor HIF1α and related bioenergetics changes (Warburg effect) play an initiating role in glucotoxicity of metabolic disorders [4,5,6].

The hypoxia inducible factor 1 (HIF1), is a transcription factor that is a crucial element for cell adaptation to hypoxia [7,8,9]. In low oxygen conditions, cells switch their energy metabolism to anaerobic glycolysis to avoid reduction of energy production. This is followed by changes in cell differentiation and tissue remodeling [10]. HIF1 is a heterodimeric protein that consists of two sub-units, HIF1α and HIF1β, which are expressed constitutively, but HIF1α expression is induced by low oxygen level [11]. Moreover, under the normoxic conditions, HIF1α sub-unit is quickly degraded, in hypoxia is not, so HIF1α and HIF1β, are translocated to the nucleus, where they form a heterodimer, which binds to the hypoxia response element (HRE) of the gene promoter for transactivation [11,12]. As the key regulator of cell response to hypoxia, HIF-1 signaling manages many genes’ expression, which take part in metabolism [12,13], angiogenesis [10,14], erythropoiesis [15], and cell survival [16]. Moreover, overly active HIF1 upregulates genes related to glycolysis in normoxic conditions. This phenomenon is known as the Warburg effect [17]. This situation is characteristic for many cancer cells’ metabolism, but there are some literature data which indicate the occurrence of the Warburg effect in normal cells to protect against oxidative stress, and hyperglycemia is well-known cause of raised oxidative damage [18,19,20]. What is more, transient hypoxia is a normal mechanism for inducing placental development [21]. However, in the case of gestational diabetes, it was proposed that pregnancy hyperglycemia induces chronic tissue hypoxia in the fetus [22,23]. Probably, fetal hyperinsulinemia which develops as a reaction to maternal hyperglycemia is the reason of chronic placental hypoxia [24,25]. In the last years, it has appeared a hypothesis that the WWOX protein regulates the balance between oxidative phosphorylation and glycolysis in cells by HIF1α transactivation modulation [26].

The WW domain-containing oxidoreductase (*WWOX*) gene is located at 16q23.1-q23.2 and spans the most active common fragile sites involved in cancer, FRA16D [27]. The *WWOX* is known as a tumor suppressor gene [28]. Deletions within the WWOX coding sequence and loss of *WWOX* expression are observed in many kinds of tumors [29], for example breast [27,30,31], ovarian [32] and non-small cell lung cancers [33,34]. The gene encodes a 46-kDa protein consisting of two N-terminal WW domains and a central short-chain dehydrogenase/reductase domain [27]. The first WW domain is responsible for majority of pathways in which WWOX is involved through binding the PPxY (where P is proline, Y is a tyrosine and x is any amino acid) motifs of a partner [35]. Several WWOX protein partners have been identified, for example p73 [36], ErbB4 [37], Ap2α and γ [38,39], Runx2 [40], Dvl2 [41] and HIF1α [26], so WWOX is involved in many cellular process, including metabolism. The first reports described *Wwox*-null mice, which died by 3 weeks after birth and demonstrated defects in growth rate and steroid metabolism as a consequence of reduced serum calcium, hypoproteinuria and hypoglycemia [42]. Further research showed that WWOX is involved in high-density lipoprotein (HDL) cholesterol, triglyceride (TG) and general lipoprotein metabolism [43], and also glucose metabolism regulation by HIF1α modulation [26]. Under aerobic conditions, *WWOX* deficiency was found to be associated with increased expression of genes coding glycolytic enzymes, including *HK2, PKM2, LDHA*, and switches pyruvate away from the mitochondrial tricarboxylic acid (TCA) cycle. Therefore, in normal cells and normoxia WWOX inhibits aerobic glycolysis and enhances the mitochondrial Krebs cycle by interaction (sequestration) with HIF1α, the master regulator of glycolytic genes [26,44]. Additionally, WWOX plays an important part in controlling glucose homeostasis in skeletal muscles and its absence leads to reduced mitochondrial oxidation, enhanced glycolysis, and fatty acid oxidation, which can lead to the development of metabolic syndrome [45]. Further, it is observed a significant association between *WWOX* variants and diabetes [45,46,47,48]. The genome-wide association study of type 2 diabetes (T2DM) in Han Chinese population showed among others that genetic variant near *WWOX* gene (rs7192960) is associated with reduced insulin secretion [46]. In quantitative trait analyses, *WWOX* rs17797882 was associated with decreased HOMA-β (β-cell function indicator) in the Han Chinese population [47], which was not confirmed by a study on the Japanese population [48]. What is more, Aqeilan et al. analyzed genome-wide association studies (GWAS) from the Type 2 Diabetes Knowledge Portal website and demonstrated that several *WWOX* variants are associated with metabolic syndrome disorders including type II diabetes [45].

The response of *WWOX* KO cells and tissues, including skin fibroblasts, to conditions of hyperglycemia, and hyperglycemia in conjunction with hypoxia is unknown. A better understanding of the molecular response to hypoxia and hyperglycemia of fibroblasts cells with altered *WWOX* expression can lead to improving knowledge of diabetes. Especially due to the fact that fibroblasts are the most abundant cell type in connective tissue and are involved in producing and remodeling the extracellular matrix [49].

Our previous study showed decreased *WWOX* expression and an increase of *HIF1A* and its targets glycolysis genes in gestational diabetes mellitus (GDM) patients in comparison to control [50]. These results indicated that WWOX modulates HIF1α activity in GDM patients. The goal of this study was a more in-depth understanding of the mechanisms behind this relationship in hyperglycemia and hypoxia. To investigate this an in vitro experiment in the human fibroblast cell line was designed and conducted. Relatively little is known about HIF1α participation in the pathogenesis of diabetes. Most of the research is concerned with the diabetes pathological complications. What is more, as one of the first we focus on the share of the *WWOX/HIF1A* axis in diabetes.

Accordingly, we silenced or overexpressed *WWOX* expression in the 1BR.3.N human skin fibroblasts. The changes in glycolysis were assessed by measuring the mRNA and protein expression of glycolytic enzymes and their enzymatic activity. 

## 2. Results

### 2.1. Stable Transfection Confirmation

**Figure 1:**
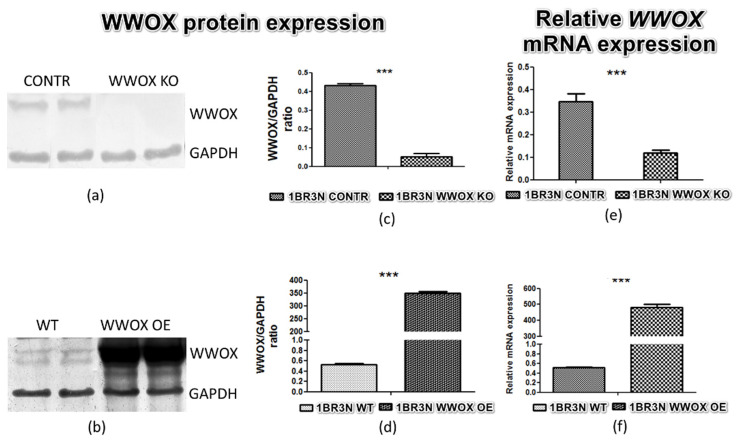
Confirmation of 1BR.3.N cell line transfection on the protein level (**a**,**b**), calculated relative protein expression (OD WWOX/OD GAPDH) (**c**,**d**) and relative *WWOX* mRNA expression (**e**,**f**). The values are mean ± SEM. ***, *p* < 0.001.

Both increase and decrease of WWOX protein (Figure 1a–d) and mRNA (Figure 1e,f) expression were identified in the 1BR.3.N cell line. The fold change in WWOX protein level between transfection variants *WWOX* KO and CONTR was about 8.5 times and between *WWOX* OE and WT more than 700. The relative expression level of WWOX mRNA was three times lower in 1BR.3.N *WWOX* KO variant in comparison to CONTR, and 850 times higher in 1BR.3.N *WWOX* OE than WT. All results are statistically significant (*p* < 0.05).

### 2.2. Hypoxia Response Element—Luciferase Reporter Assay

**Figure 2:**
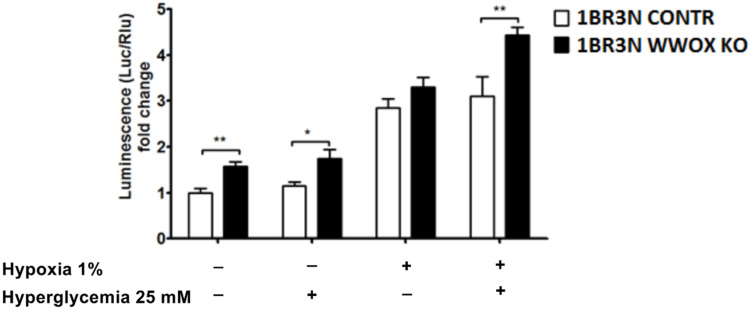
Obtained variants of 1BR.3.N cell line (1BR.3.N CONTR and 1BR.3.N *WWOX* KO) was transfected with a luciferase reporter gene under the regulation of Hypoxia-Responsive Elements (HRE) and subjected to all tested conditions for 6 h. Extracts were analyzed for luciferase activity. The values are mean ± SEM. *, *p* < 0.05; **, *p* < 0.01.

Based on the available literature data, we hypothesized that WWOX has an influence on HIF1α activity to recognize hypoxia response element (HRE) in the human fibroblast cell line. It was examined the effect of *WWOX* expression on the activity of a luciferase reporter carrying multiple hypoxia-responsive elements (HREs) under all tested conditions to check whether WWOX directly disturbs HIF1α transactivation function. HIF1α activity in all obtained conditions was quantified using a luminescence assay. Results showed a statistically significant increase in HIF1α activity in the case of the 1BR.3.N *WWOX* KO transductant compared to the 1BR.3.N CONTR under the conditions of normoxia normoglycemia (*p* < 0.01), normoxia hyperglycemia (*p* < 0.05) and hypoxia hyperglycemia (*p* < 0.01) (Figure 2). It was also confirmed that 1BR.3.N *WWOX* overexpression cells showed a significant reduction in HIF1α activity under all tested conditions, by about 50% in normoxia normoglycemia (*p* < 0.05), by about 70% in normoxia hyperglycemia (*p* < 0.05), and almost 6.5-fold decrease in HIF1α activity under hypoxic conditions (*p* < 0.001), both in standard and elevated glucose concentration in the culture medium (Figure S1a).

### 2.3. WWOX Depending Glucose Uptake

**Figure 3:**
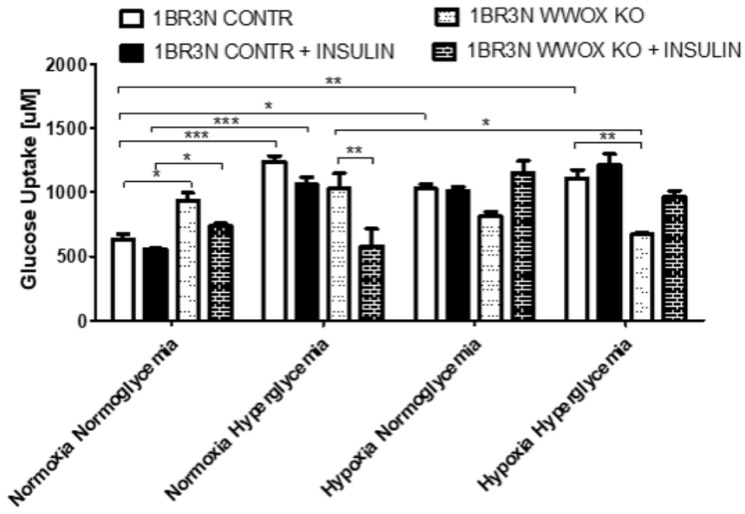
Glucose uptake in *WWOX* KO and CONTR variants of 1BR.3.N cell line incubated all tested conditions (Normoxia Normoglycemia, Normoxia Hyperglycemia, Hypoxia Normoglycemia, Hypoxia Hyperglycemia) with or without insulin for 48 h. The values are mean ± SEM. *, *p* < 0.05; **, *p* < 0.01; ***, *p* < 0.001.

Due to our main interest in the study of the influence of *WWOX* expression on glucose metabolism, in the next step, we examined whether *WWOX* expression causes differences in glucose uptake in all tested culture conditions. We observed that *WWOX* silencing significantly increased glucose uptake in normoxia normoglycemia and reduced glucose uptake in hypoxia hyperglycemia conditions in the absence of insulin. The addition of insulin results also in the increased (*p* < 0.05) glucose uptake in normoxia normoglycemia, but not in other conditions (Figure 3).

We also performed a glucose uptake assay in 1BR.3.N *WWOX* OE and WT cell variants. *WWOX* overexpression resulted in a significant (*p* < 0.05) increase of the glucose uptake in normoxia hyperglycemia, however, when fibroblasts were cultured in hypoxia, the effect of *WWOX* overexpression was inverted, resulting in the reduction of glucose uptake. Pretreated with insulin, the 1BR.3.N *WWOX* OE fibroblasts, showed a significant (*p* < 0.05) increase in glucose uptake in the normoxia hyperglycemia, but glucose uptake decrease in hypoxia normoglycemia and hyperglycemia in comparison to 1BR.3.N WT (Figure S1b).

### 2.4. Glucose Related Genes Expression—RT-qPCR Analysis

**Figure 4:**
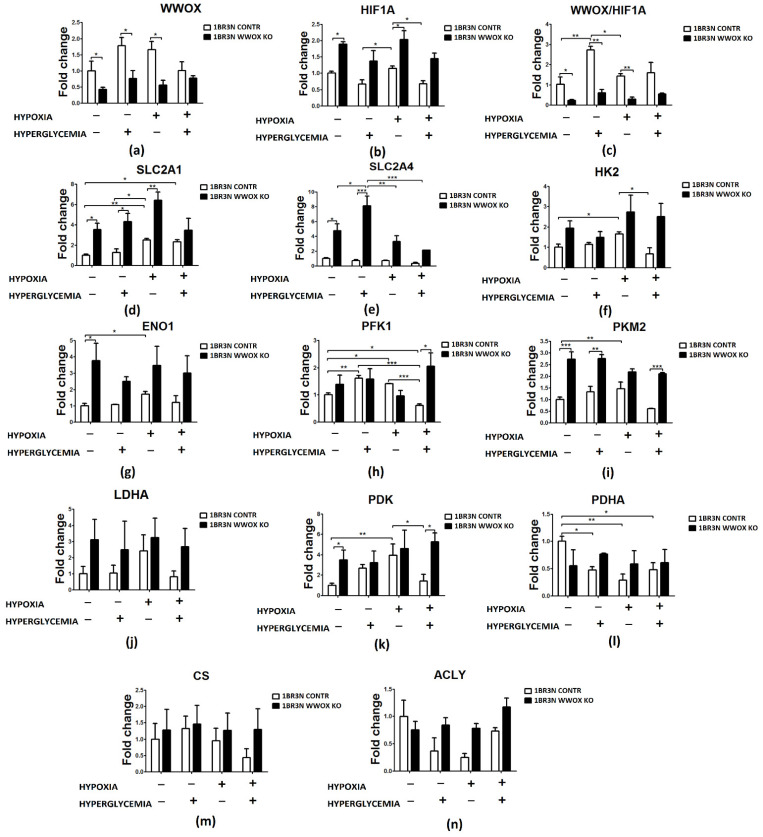
Relative expression fold change of glycolysis-associated genes detected by RT-qPCR in *WWOX*-silencing 1BR.3.N cells after 48 h incubation in four tested condition. Bar graphs showing the relative mRNA expression (the ratio of the target gene relative to the reference genes *RPS17, RPLP0, H3F3A*) of *WWOX* (**a**), *HIF1A* (**b**), the *WWOX/HIF1A* ratio (**c**) and all WWOX/HIF-related genes including those involved in glucose transport (*SLC2A1* (**d**), *SLC2A4* (**e**)), glycolytic pathway (*HK2* (**f**), *ENO1* (**g**), *PFK* (**h**), *PKM2* (**i**), *LDHA* (**j**)), *PDK* (**k**), *PDHA* (**l**), *CS* (**m**) and *ACLY* (**n**). Results of ANOVA with a post-hoc Tukey-Test are shown as mean ± SEM. *, *p* < 0.05; **, *p* < 0.01; ***, *p* < 0.001.

RT-qPCR analyses were conducted after exposure to all tested culture conditions. The *WWOX* expression was statistically significantly down-regulated in normoxia normoglycemia and hyperglycemia condition (*p* < 0.05) and in hypoxia normoglycemia (*p* < 0.05). In all tested oxygen and glucose concentration level conditions, *WWOX* silencing resulted in increasing of the expression level of HIF1A (1.9-fold for normoxia normoglycemia, *p* < 0.05; 1.9 for normoxia hyperglycemia, *p* > 0.05; 1.5-fold for hypoxia normoglycemia, *p* < 0.05 and 1.9-fold for hypoxia hyperglycemia conditions, *p* > 0.05) and examined genes associated with glucose transport (SLC2A1, SLC2A4) and glycolysis (HK2, ENO1, PKM2, PDK). Only some results are statistically significantly different (Figure 4). In normoxia normoglycemia conditions expression levels of SLC2A1, SLC2A4, ENO1, PKM2, and PDK (all *p* < 0.05) are significantly higher in the *WWOX* KO cell line than in control. In normoxia hyperglycemia we observed significantly increased expression of glucose transporters SLC2A1 (nearly fourfold, *p* < 0.05) and SLC2A4 (more than sevenfold, *p* < 0.05) and PKM2 (*p* < 0.01). We noted a significantly greater expression level of SLC2A1 (*p* < 0.05) in hypoxia normoglycemia conditions. Hypoxia hyperglycemia was characterized by upregulation of some tested genes’ expression levels after *WWOX* downregulation. We detected statistically significant different in expression level of PFK1 (3-times; *p* < 0.05) and PKM2 (nearly 4-times; *p* < 0.001). When it comes to examined genes associated with the tricarboxylic acid (TCA) cycle (PDHA, CS, ACLY), in hypoxia normoglycemia conditions ACLY upregulation was detected (*p* < 0.05).

On the other hand, as shown in Figure S2, the expression of *SLC2A4* (*p* < 0.05) were downregulated and *ENO1* (*p* < 0.05), *PDK* (*p* > 0.05) and *PDHA* (*p* > 0.05) expression was upregulated in variants representing *WWOX* overexpression in normoxia normoglycemia condition. In normoxia hyperglycemia, in the case of *WWOX* overexpression, the *PDHA* gene expression increased 3-times (*p* < 0.05) compared to the appropriate control. Furthermore, the expression of *ENO1* (*p* < 0.01) was raised in *WWOX* overexpression cases in hypoxia normoglycemia. The *ENO1* gene expression was lower (*p* < 0.001) and CS (*p* < 0.05) was higher in cells with the 1BR.3.N *WWOX* OE in comparison to control in hypoxia hyperglycemia. 

### 2.5. Anaerobic Glycolysis Metabolite and Metabolic Enzymes Affected by WWOX Silencing

The analysis of enzyme kinetics revealed differential activities of some glycolytic enzymes in the lysates of 1BR.3.N *WWOX* KO cell lines compared with control cells depending on the cell culture conditions. In particular, the activities of hexokinase (HK), lactate dehydrogenase (LDHA), and lactate concentration in the case of normoxia normoglycemia conditions were statistically increased in the *WWOX* KO line, while the activities of pyruvate dehydrogenase kinase (PDK) and citrate synthase (CS) were not changed. Of these, the single most significant and greatest change in activity was observed for hexokinase, whose activity in 1BR.3.N *WWOX* KO cells was 1,6-fold higher than in control empty vector cells (*p* < 0.01). Lactate dehydrogenase activity was 1,4-fold greater (*p* < 0.01) and lactate concentration in culture medium was 1,6-fold higher (*p* < 0.01) than in control. 

In normoxia hyperglycemia conditions, lactate dehydrogenase activity (*p* < 0.01) and lactate level (*p* < 0.001) was still upregulated in 1BR.3.N *WWOX* KO cells in comparison to 1BR.3.N CONTR but did not contribute to the changes in hexokinase, pyruvate dehydrogenase, and citrate synthase activity under these conditions. 

In hypoxia normoglycemia, 1BR.3.N *WWOX* KO cell line was characterized by glycolysis enhanced through hexokinase activity upregulation (*p* < 0.001) and then by the growth of lactate concentration (*p* < 0.001). We didn’t observed any significantly important differences in the activity of pyruvate dehydrogenase, lactate dehydrogenase, and citrate synthase. 

Lactate concentration in medium was also 1,3-higher (*p* < 0.01) in hypoxia hyperglycemia. *WWOX* silencing exhibited no significant effect on enzymes activity in hypoxia hyperglycemia culture conditions.

**Figure 5:**
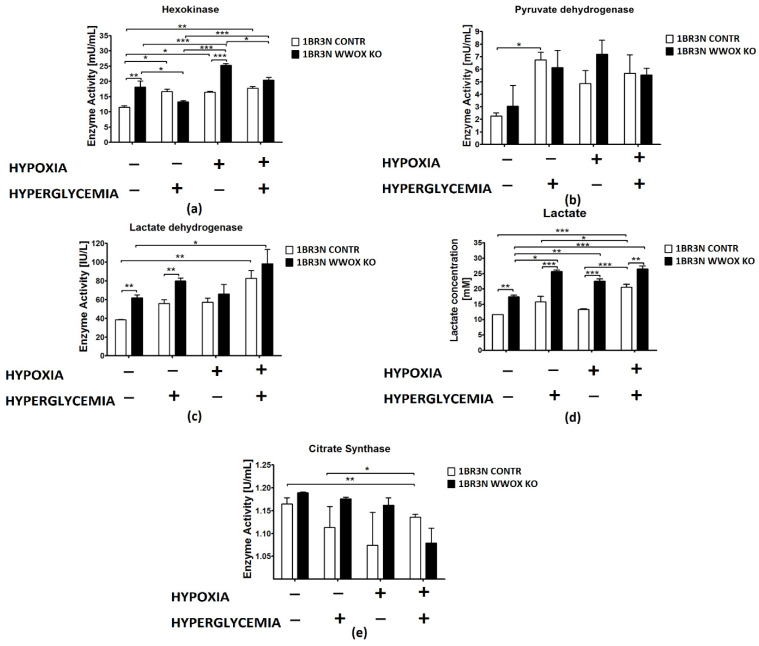
Colorimetric method study of aerobic glycolysis metabolic enzymes and metabolites in 1BR.3.N CONTR and *WWOX* KO cells after 48 h incubation in four tested condition. Bar graphs showing the enzyme activity of Hexokinase (**a**), Pyruvate Dehydrogenase (**b**), Lactate Dehydrogenase (**c**) Citrate Synthase (**e**) and Lactate concentration (**d**). Results of ANOVA with a post-hoc Tukey-Test are shown as mean ± SEM. *, *p* < 0.05; **, *p* < 0.01; ***, *p* < 0.001.

All results were shown in Figure 5.

Comparing with RT-qPCR results, we didn’t find many significantly important differences in enzymes activity between 1BR.3.N WT and 1BR.3.N *WWOX* OE cell lines. *WWOX* overexpression induced downregulation lactate dehydrogenase activity (*p* < 0.01) in hypoxia hyperglycemia and pyruvate dehydrogenase activity (*p* < 0.001) decrease in hypoxia normo- and hyperglycemia. Lactate concentration were significantly higher in case of *WWOX* overexpression in comparison to 1BR.3.N WT cell line in normoxia normoglycemia and hypoxia hyperglycemia (Figure S3).

### 2.6. Protein Analyses by Western Blot

#### 2.6.1. Cytoplasmic and Nuclear Distribution of WWOX and HIF1α Proteins 

**Figure 6:**
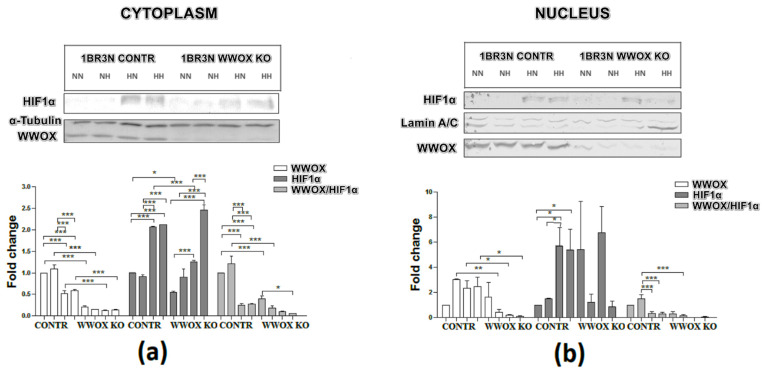
WWOX and HIF1α western blot analysis of cytoplasmic (**a**) and nuclear (**b**) fractions of CONTR and *WWOX* KO 1BR.3.N cells after 48 h incubation in four tested condition. Results of ANOVA with a post-hoc Tukey-Test are shown as mean ± SEM. *, *p* < 0.05; **, *p* < 0.01; ***, *p* < 0.001; NN—normoxia normoglycemia, NH—normoxia hyperglycemia, HN—hypoxia normoglycemia, HH—hypoxia hyperglycemia.

The western blot analyses found that the level of the WWOX protein is significantly depleted in a cytoplasmic fraction in all tested conditions in case 1BR.3.N *WWOX* KO in comparison to CONTR (more than 4-fold for normoxia normo- and hyperglycemia, both *p* < 0.001; 3-fold for hypoxia normo- and hyperglycemia conditions, both *p* < 0.01). We observed WWOX expression also in nuclear fraction. In normoxia hyperglycemia, hypoxia normo- and hyperglycemia, WWOX protein is significantly lower also in nucleus (all *p* < 0.05). To check that WWOX can have an influence on HIF1α protein, we analyze HIF1α protein level in the cytoplasm and nucleic fraction after exposure to all tested conditions. Compared with controls, the expression of HIF1α protein was significantly decreased in 1BR.3.N *WWOX* KO in comparison to CONTR in normoxia normoglycemia (2-fold; *p* < 0.01) and hypoxia normoglycemia (nearly 2-fold; *p* < 0.001) in cytoplasm fraction. We didn’t observed WWOX influence on HIF1α protein in nuclear fraction. Additionally, hypoxia (normoglycemia and hyperglycemia) condition decreased WWOX protein expression in cytoplasm relative to normoxia (normoglycemia and hyperglycemia) in 1BR.3.N CONTR variant. One repetition is shown in the Figure 6.

Additionally, it was observed that 1BR.3.N *WWOX* OE cell line variant demonstrated reduction of PKM2 and LDHA protein expression in normoxia normoglycemia, decrease of HK2 protein in normoxia hyperglycemia, and PKM2 reduction in hypoxia hyperglycemia condition relative to 1BR.3.N WT cell line variant (Figure S4).

#### 2.6.2. Glucose Transporter and Glycolysis Proteins Western Blot Analyses

**Figure 7:**
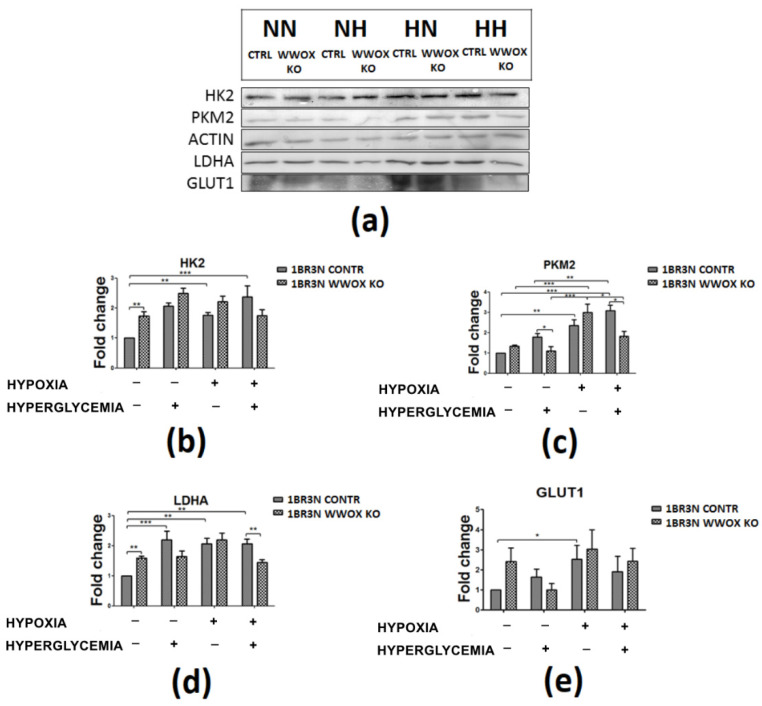
The western blot analysis of selected proteins (HK2, PKM2, LDHA, GLUT1) of CONTR and *WWOX* KO 1BR.3.N cells after 48h incubation in four tested condition (**a**). Bar graphs showing the relative level OD density of HK2 (**b**), PKM2 (**c**), LDHA (**d**), GLUT1 (**e**) to ACTIN. Results of ANOVA with a post-hoc Tukey-Test are shown as mean ± SEM. *, *p* < 0.05; **, *p* < 0.01; ***, *p* < 0.001. NN—normoxia normoglycemia, NH—normoxia hyperglycemia, HN—hypoxia normoglycemia, HH—hypoxia hyperglycemia.

Western blot was performed to assess the expression of HK2, PKM2, LDHA and GLUT1 at a protein level in all obtained cell line variant in tested conditions. The present study demonstrated that 1BR.3.N *WWOX* KO variant exhibits higher HK2 (*p* < 0.01) and LDHA (*p* < 0.01) protein expression in normoxia normoglycemia, lower PKM2 (*p* < 0.05) in normoxia hyperglycemia, and lower PKM2 (*p* < 0.05) and LDHA (*p* < 0.01) in hypoxia hyperglycemia in comparison to 1BR.3.N CONTR (One repetition is shown in the Figure 7).

Additionally, we observed in 1BR.3.N *WWOX* OE cell line variant the reduction of PKM2 and LDHA protein expression in normoxia normoglycemia, the decrease of HK2 protein in normoxia hyperglycemia, and PKM2 reduction in hypoxia hyperglycemia condition relative to 1BR.3.N WT cell line variant (Figure S5).

### 2.7. Immunocytochemistry of WWOX and HIF1α

**Figure 8:**
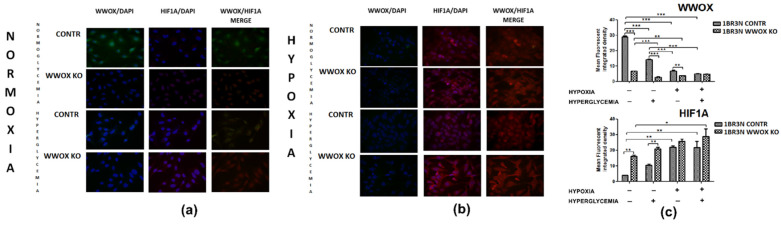
Immunocytochemistry analysis of WWOX and HIF1α in 1BR.3.N CONTR and WWOX KO cells after 6h incubation in normoxia (**a**) and hypoxia (**b**). Calculation of Total Corrected Cell Fluorescence (TCCF) (**c**). Results of ANOVA with a post-hoc Tukey-Test are shown as mean ± SEM. *, *p* < 0.05; **, *p* < 0.01; ***, *p* < 0.001.

The immunocytochemistry confirmed downregulation of *WWOX* by transfection in normoxia normoglycemia (*p* < 0.001) and this effect was maintained in normoxia hyperglycemia (*p* < 0.001) and hypoxia normoglycemia (*p* < 0.01). Hypoxia hyperglycemia cultivation of 1BR.3.N *WWOX* KO cells did not modify the total intracellular content of WWOX compared with 1BR.3.N CONTR (Figure 8). *WWOX* downregulation resulted in HIF1α increase in normoxia normoglycemia and hyperglycemia condition (both *p* < 0.01). It was not found statistically significant WWOX reduction influence on total intracellular content of HIF1α protein in hypoxia normo- and hyperglycemia condition (Figure 8). The results for comparison 1BR.3.N *WWOX* OE with 1BR.3.N WT in all tested conditions were shown in Figure S6.

## 3. Discussion

The WWOX protein has been identified as a glucose metabolism regulator via HIF1α modulation [26]. The WWOX is capable of sequestering HIF1α in the cytoplasm, therefore inhibiting the HIF1α transactivation function and represing the expression of the glycolytic enzymes [26]. Our previous study suggests a significant contribution of the *WWOX* gene to glucose metabolism in patients with gestational diabetes mellitus. We showed decreased *WWOX* expression in GDM compared to normal pregnancy, and what is most important a significant reduction of *WWOX/HIF1A* ratio [50]. These results indicated that WWOX modulates HIF1α activity and the effect of HIF1α excessive activation was to increase the expression of genes encoding proteins directly involved in the glycolysis. It might lead to pathological changes in glucose metabolism observed in gestational diabetes. The goal of this study was to analyze the impact of WWOX on glycolysis in hyperglycemia and hypoxia conditions in a human fibroblast cell line, based on an assumption of the importance of the *WWOX* gene in the pathophysiology of diabetes [45,47,50,51,52]. We determined that knock-out of *WWOX* leads to increased *HIF1A* mRNA and protein level together with its translocation to the nucleus and raise of its transactivation function. Therefore, *HIF1A* upregulation leads to an increase in the expression of all studied genes encoding glycolysis proteins. We observed the significantly diverse expression of *SLC2A1, ENO1, PKM2, PDK*. These results correspond to currently published reports on the effects of WWOX on HIF1A and its target genes. Aqeilan et al. observed that Wwox-deficient mouse embryonic fibroblasts (MEFs) exhibited increased HIF1α protein levels and its activity. These cells displayed also an increase in *SLC2A1, HK2, PKM2, LDHA*, and *PDK* expression, and glucose uptake. However, in their results, *HIF1A* mRNA levels are comparable between *WWOX* KO and WT skeletal muscles of mice [26]. Additionally, in our experiment also *SLC2A4* glucose transporter was increased. We have shown that glucose uptake, both insulin-dependent and independent, was upregulated in *WWOX* KO cells. Furthermore, in *WWOX* deficient cells, we recognized the increase of hexokinase II and lactate dehydrogenase A proteins concentrations and the raise of these enzymes activities. This clearly indicates that silencing of *WWOX* expression in the human fibroblast line 1BR.3.N leads to a shift towards anaerobic glycolysis under normoxic conditions, i.e., the Warburg effect [53,54]. As Iacobini noted, despite enhanced glucose uptake and glycolysis dysfunction having long been described in diabetic tissues, the Warburg effect role in diabetes was not proposed for a long time [55]. This is probably due to the fact that has not still been clarified what is the pathophysiological meaning of these changes and what are the early instigators of the Warburg effect in diabetes. Recent research reports suggested that abnormal HK2 increased activity plays a key role when control of glycolysis is impaired, as in the case of diabetes. This leads to an abnormal increase in glycolytic intermediates, which play role in the tissue-specific chronic pathogenesis of hyperglycemia associated with diabetes [56].

As there is growing evidence that mitochondrial superoxide production is dispensable in the pathogenesis of diabetes, it was suggested that stabilization of HIF1α and associated metabolic reprogramming, resembled the Warburg effect, were the initial events in the cellular biochemical abnormalities induced by glucose in diabetes [4,57]. Therefore, the *WWOX/HIF1A* lowered expression ratio should be considered as a molecular risk factor for diabetes type 2 and gestational as we already reported [50]. It is worth noting that XIAP, a member of the inhibitors of apoptosis proteins (IAPs) [58] is a regulator of HIF1α polyubiquitination [59]. It has been found that XIAP promotes HIF1α nuclear retention leading to an increase in the expression of HIF1 target genes [59]. It is worth mentioning that the elevated formation of new *β*-cells in T2DM in relation to non-diabetic adults, has no effect in in the number of cells secreting insulin due to increased apoptosis [60]. XIAP gene expression may protect *β*-cells and human islets from apoptotic cell death [61], so the relationship between *WWOX/HIF1A* axis with XIAP can be further investigated in a broader context with the possibility of using it in the prevention and treatment of insulin secretion disorders.

**Figure 9:**
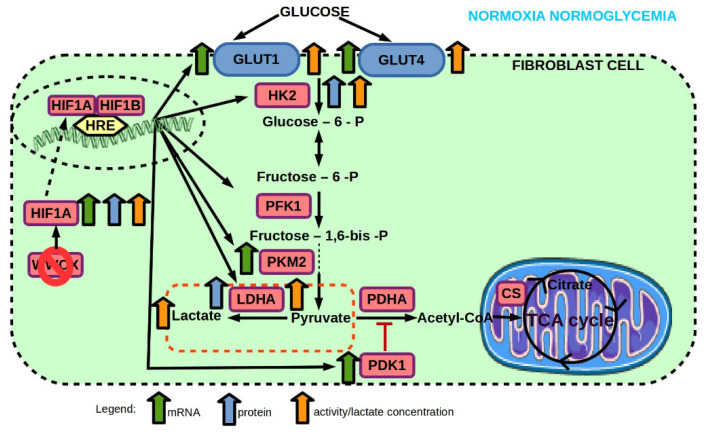
Effect of WWOX KO on mRNA and protein expression, and enzyme activity of genes involved in glucose transport and glycolysis pathway of 1BR.3.N fibroblast cell line in normoxia normoglycemia condition. Based on free images from Servier Medical Art; CC BY 3.0.

Contrary to the enhancement of the glycolysis pathway and the increase of lactate as the result, we did not observe any consecutive changes in citrate synthase activity in WWOX depleted cells. Citrate synthase is a key mitochondrial enzyme that is commonly used as a quantitative marker for the content of intact mitochondria and its activity is reduced in muscles of type 2 diabetic patients [62,63]. However, several literature reports discuss changes in the expression and activity of citrate synthase under the *HIF1A* influence but have not shown unequivocal results [64,65]. It has been observed that citrate synthase activity remaining significantly increased in hypoxic diabetic rats compared with hypoxic controls [64]. However, in another human study, citrate synthase activity in type 2 diabetic patients and control subjects was comparable [65]. Under hypoxia, HIF1α switches glucose metabolism from prevailing oxidative phosphorylation to anaerobic glycolysis by simultaneously increasing the expression of glycolytic enzymes and restraining mitochondrial function and oxygen consumption [4,66]. The *WWOX*-deficient mice exhibited a reduction in oxygen consumption and amount of the Krebs cycle intermediates [26], as well as the *Drosophila melanogaster* model, which showed reduced mitochondrial respiration by decreasing expression of any one of the six mitochondrial respiratory complex genes, so *WWOX* deficiency affect mitochondrial function [67]. Nonetheless, in our study, citrate synthase expression and activity were tested and we did not observe WWOX influence on it. However, in *WWOX* deficient human fibroblasts we detected elevated protein concentration and activity of lactate dehydrogenase as well as increased expression of *PDK1*—the PDHA inhibitor—an essential link between glycolysis and the TCA cycle (Figure 9). 

In type 2 diabetes, multiple aspects of glucose homeostasis are disturbed which leads to hyperglycemia [68,69]. These hyperglycemic conditions can overproduce NADH and lower nicotinamide adenine dinucleotide (NAD), thereby creating NADH/NAD redox imbalance leading to cellular pseudohypoxia [2]. Thus, we analyzed the hyperglycemia influence on control human fibroblasts in normoxia. We showed that *HIF1A* expression, its protein’s localization and transactivation function didn’t change in response to hyperglycemia in the control normal cell line, despite the reduction of WWOX protein. In control cells, we recognized an increase in glucose uptake, a raise of hexokinase and pyruvate dehydrogenase activity, and LDHA protein accumulation under hyperglycemia. We also detected changes in mRNA expression, like *PFK1* increase and *PDHA* decrease. 

Hyperglycemia increases the flow of glucose into the cell through the so-called mass effect [68,70]. In our investigation and as it was already reported, hyperglycemia stimulates glucose uptake by increasing the activity of its transporters on cell surface membranes [70]. The greater amount of available glucose in the examined fibroblasts further led to an increase of hexokinase and pyruvate kinase activities with the raise of lactate production but without changes in citrate synthase expression and activity.

In *WWOX* KO fibroblasts, we found that the *WWOX/HIF1A* ratio was lower on mRNA and protein levels, both in cytoplasm and nucleus localization. It should be noted, that despite elevated glucose levels in media, examined cells did not show elevated glucose uptake. Nonetheless, we observed the enhancement of glycolysis leading to the increased amount of lactate in response to high glucose concentration. We noted the increase of HIF1α total protein amount and raise of its transactivation function. We detected the mRNA expression growth of its targets genes: *SLC2A1* and *PKM2*. We also observed the increased *SLC2A4* expression. Although HIF1α is not a direct regulator of the *SLC2A4* glucose transporter, there are reports showing a correlation between *HIF1A* expression and its activity [71]. In skeletal muscle cells, HIF1α loss impairs GLUT4 translocation and glucose uptake [71]. We did not recognize the simultaneous increase in the amount of protein and enzymatic activity of studied elements of the glucose transport and glycolysis pathway in hyperglycemia, which was present in normoglycemia. What is more critical, in our observations of *WWOX* KO fibroblasts in comparison to control in normoxia hyperglycemia, we stated that the *SLC2A1* and *SLC2A4* mRNA increase does not translate into protein level and glucose uptake growth. Despite the lack of differences in total glucose uptake between *WWOX* KO and CONTR, we found a significant 2-time reduction under insulin addition, suggesting a WWOX role in maintaining insulin sensitivity in fibroblasts. Our data demonstrated that hyperglycemia is insufficient to induce insulin resistance in the control fibroblast cell line. In all tested conditions, we did not observe significant differences between insulin-dependent and independent glucose uptake in control cells, which is consistent with the available literature results which indicate that normal fibroblasts show little metabolic response to insulin [72,73]. Because of these reasons, we have not conducted further studies influenced by insulin, but it should be under consideration. No in vivo models of fibroblast-specific resistance to insulin is currently described, but there has been evidence supporting an insulin resistance role in fibroblast dysfunction [74]. The glucose uptake by fibroblasts in obese patients with type II diabetes has been increased by 50% compared to the healthy control, while insulin-dependent glucose uptake was reduced by as much as 3-fold [74]. 

Additionally, glucose transporters’ expression and activity correlates with reactive oxygen species production (ROS). In skeletal muscle cells, a decrease of GLUT1-mediated glucose transport causes increased = ROS, whereas an increased glucose transport would result in decreased ROS levels [75]. Unlike that, the proliferating keloid fibroblasts (KFs) and showed that GLUT1 expression and ROS levels were increased in keloid tissue compared to normal fibroblasts [76]. GLUT1 inhibition suppressed the ROS levels in KFs [76]. This was not directly investigated but it is worth mentioning that oxidative stress plays a role in the pathogenesis and development of diabetes complications [77]. High glucose concentration induces ROS formation, inflammation, and enhances oxidative stress [78,79,80,81]. Hyperglycemia impairs the endogenous antioxidant defense system in patients with diabetes [77]. Excess ROS impairs dermal fibroblast proliferation and migration [82] and perturbations in ROS have been linked to Warburg effect [83]. ROS generation has been implicated in the stabilization and transcriptional activation of HIF1α [84].Moreover, WWOX, through its C-terminal SDR domain, regulates the ROS generation in *Drosophila* [85] and mammalian cells [86]. It can be interesting to continue our work in this direction mainly due to evidence which have proven the utility of antioxidants which might therefore be helpful for treating diabetes and its complication [77]. However, there is a report indicating that ablation of intracellular ROS by antioxidants failed to alleviate hypoxia-induced aberrant production of adiponectin in adipocytes [87]. Low plasma levels of the anti-inflammatory adipokine adiponectin is associated with obesity-related insulin resistance [88] and correlates negatively with hemoglobin A1c [89] and HOMA-IR [90] in T2D patients. For these reasons, WWOX targeting may prove to be more effective. Leptin expression, another one of adipocytokines, controlling insulin-dependent glucose transport to adipocytes [91] was found to be hypoxia-inducible [92], which is stimulated in adipose tissue [93] and placenta [94] through HIF1. Hypoxia-inducible factor is also responsible for upregulation of visfatin mRNA expression [95] in the fat tissue of obesity patients and significantly higher levels of visfatin was observed in the population of obese T2DM patients compared to healthy subjects [96]. These reports indicate that it is worth including these factors in the future research on the significance of *WWOX/HIF1A* axis in metabolic disorders, knowing also that WWOX disruption alters HDL and lipoprotein metabolism [43].

**Figure 10:**
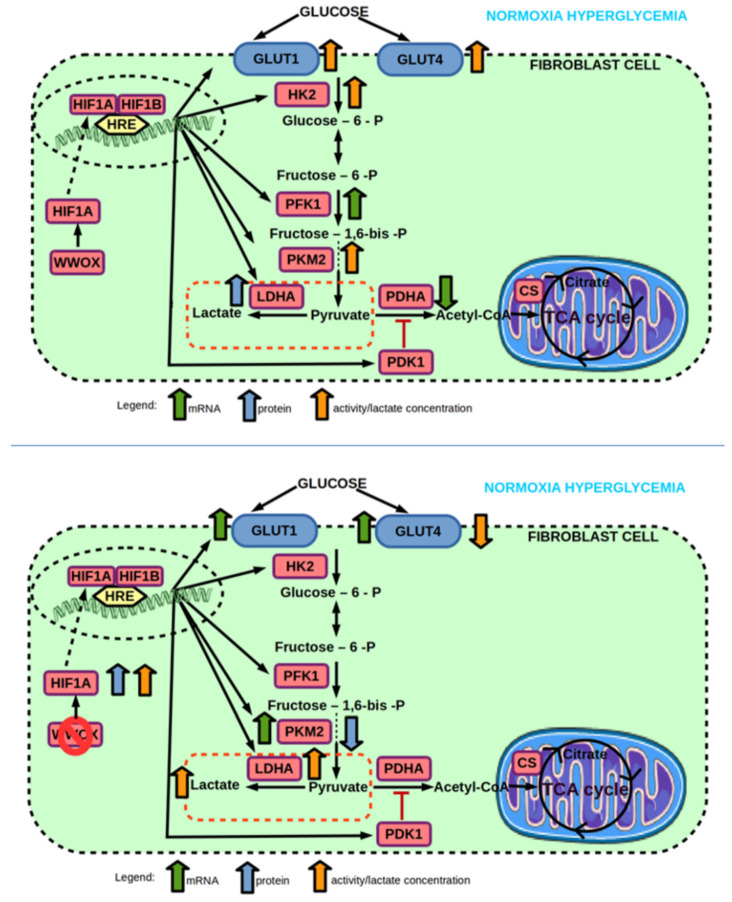
(**top**) Effect of hyperglycemia on mRNA and protein expression, and enzyme activity of genes involved in glucose transport and glycolysis pathway of 1BR.3.N fibroblast cell line in normoxia. (**bottom**) Effect of *WWOX* KO on mRNA and protein expression, and enzyme activity of genes involved in glucose transport and glycolysis pathway of 1BR.3.N fibroblast cell line in normoxia hyperglycemia condition. Based on free images from Servier Medical Art; CC BY 3.0.

Additionally, previous studies have shown that human fibroblasts, from both a healthy donor and a diabetic patient, showed an increased lactate production with a simultaneous decrease in oxygen metabolism without changes in the amount of mitochondria at an increasing glucose concentration [97]. The *WWOX* KO cells, when compared with control fibroblasts, showed a significantly increased activity of lactate dehydrogenase and the amount of lactate, indicating enhanced anaerobic metabolism in normoxia hyperglycemia conditions. Nonetheless, we did not find significant differences in the citrate synthase activity between the *WWOX* KO and CONTR variants in hyperglycemia. In addition, we did not observe meaningful changes of citrate synthase activity under the influence of hyperglycemia, both in control and *WWOX* KO cells. Citrate synthase is not only used by researchers as a biomarker of mitochondrial function [62,63] but there are even studies suggesting that hereditary citrate synthase dysfunction in skeletal muscle may be responsible for the pathogenesis of insulin resistance and diabetes [98,99]. The citrate synthase activity was lower in myotubules of diabetic patients than in the control group and what is more, insulin incubation caused an increase in its activity in healthy people, both with normal weight and obese, but it was not observed in diabetic patients [98]. There are no similar reports on human fibroblasts, but other authors have found no differences in citrate synthase activity as an effect of culturing normal fibroblasts under hyperglycemic conditions, which is in line with our results [100]. A scheme of results in normoxia hyperglycemia was shown in Figure 10.

Furthermore, in normoxia hyperglycemia, our results showed that *WWOX* over-expression stabilizes high HIF1α protein amount without changes in its mRNA expression and protein localization. We assumed that an increase in the total amount of HIF1α protein may act as a compensatory mechanism in response to increased *WWOX* expression, given its role as a modulator of the HIF1α transactivation function [26]. Interestingly, we detected HIF1α transactivation function depreciation without significant mRNA expression changes of HIF1α target genes. We can explain this by the fact that we observed low HIF1α protein level in normoxia conditions and it is known that HIF1α is mainly responsible for the control of its target genes transcriptions in hypoxia. Aqeilan et al. received similar results [26]. What is also important, our results confirmed that WWOX can be responsible for maintaining glucose flow in fibroblast cells exposed to high glucose levels. We observed a significant increase in insulin-dependent and insulin-independent glucose uptake in the case of *WWOX* overexpression in comparison to the WT variant, which is probably independent of HIF1α regulation. Other authors have proposed a mechanism that elevated levels of *WWOX* in cells mimic the poor energetic status, resulting in AMP-activated protein kinase activation [45]. AMPK is known for glucose uptake stimulation by provoking GLUT4 translocation from intracellular membranes to the cell surface [101,102,103]. Probably, GLUT1 is also activated by mechanisms stimulated by AMPK [104,105,106].

Then, hyperglycemia induces cellular hypoxia, and this hyperglycemia coupled with hypoxia may be involved in the pathogenesis of diabetes [6,49,107,108]. Therefore we examined how control cells respond to elevated sugar levels under hypoxic conditions, and then how this process was disrupted by a *WWOX* downregulation. In our hypoxia model, hyperglycemia does not induce changes in the glucose uptake and glycolytic pathway in normal hypoxic human fibroblasts. However, we observed the formation of an increased amount of lactate.

**Figure 11:**
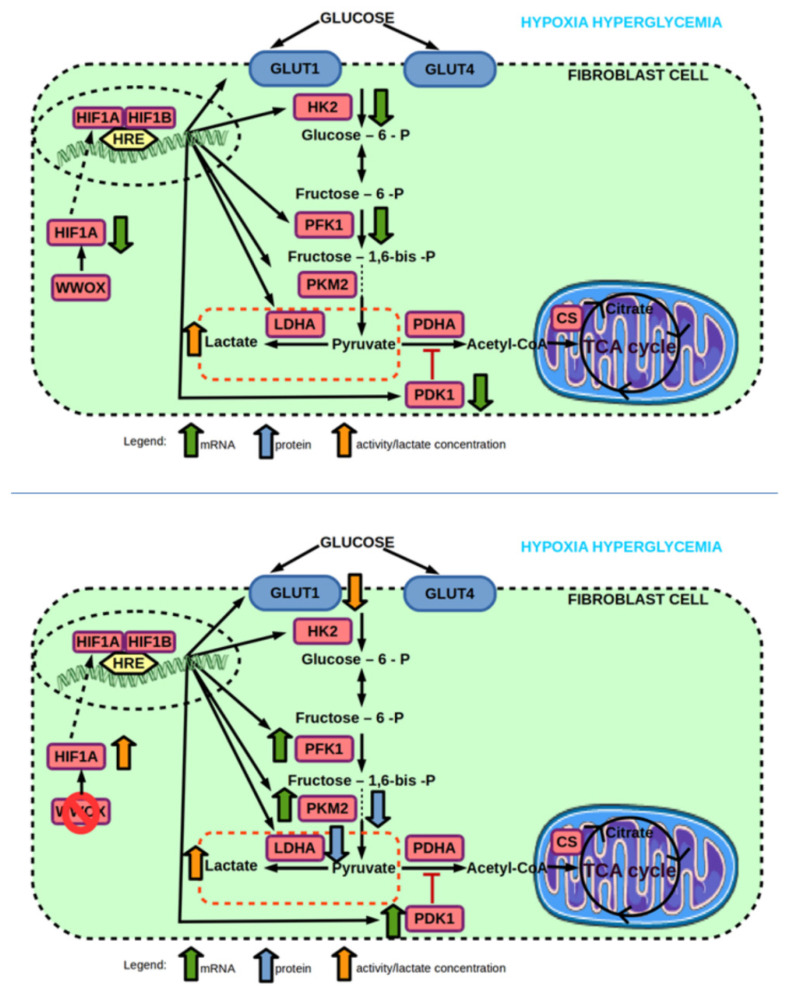
(**top**) Effect of hyperglycemia on mRNA and protein expression, and enzyme activity of genes involved in glucose transport and glycolysis pathway of 1BR.3.N fibroblast cell line in hypoxia. (**bottom**) Effect of WWOX KO on mRNA and protein expression, and enzyme activity of genes involved in glucose transport and glycolysis pathway of 1BR.3.N fibroblast cell line in hypoxia hyperglycemia condition. Based on free images from Servier Medical Art; CC BY 3.0.

Moreover, in the 1BR.3.N *WWOX* KO cell line we recognized the increase of HIF1α transactivation function in hypoxia hyperglycemia condition. Then, we observed the rise of its targets genes *PFK, PKM2*, and *PDK* mRNA expression and a decrease in glucose uptake without changes in insulin-dependent uptake, with a simultaneous increase in lactate concentration (Figure 11). We found no changes in the activity of the glycolysis enzymes. In most kinds of cells, glycolysis is activated in diabetes [109], but unlike aerobic glycolysis, relatively few is information about changes in anaerobic glycolysis in diabetes [110]. In diabetic patients, a marked increase in the lactate dehydrogenase activity [111,112] and the amount of lactate leading to local acidification [113,114,115,116] is observed compared to the healthy control group. Earlier results approved that increased lactate levels are an independent risk factor for the T2DM development [110,117], but further data suggests that lactate is not enough to induce insulin resistance in humans [118]. Additionally, intracellular lactate- and pyruvate-interconversion rates are largely increased in T2DM patients, probably due to accompanying impairment in the oxidative pathway of glucose metabolism [110]. What is more, lactate itself can downregulate the activity of glycolytic enzymes, such as hexokinase and phosphofructokinase [119]. Hypoxia is greatly associated with human physiology and pathophysiology, with a critical HIF1α function in these processes, but the role of HIF1α in the pathogenesis of diabetes is not obvious. There are various results showing weaken [1] or enhanced [120,121] HIF-1 pathway under hyperglycemia.

The obtained results are also interesting in the view that HIF1 α is an essential element in host-microbial crosstalk, which supports gut homeostasis [122]. More and more current results showed that the gut microbiome probably takes part in inflammation associated with type 2 diabetes development and therapy targeting the gut microbiome may be a likely a treatment of type 2 diabetes mellitus [123]. What is more, SNP rs9927590 (found within *WWOX* gene) has been reported to be involved in obesity and was associated with a metabolic pathway involved in short-chain fatty acid (SCFA) production [124]. The gut bacterial production of SCFA may prevent T2D through appetite control and energy homeostasis [125,126]. Additionally, butyrate, one of SCFA, has been displayed to have an influence on gut hypoxia by mitochondrial beta-oxidation and by interacting with HIF1α to maintain tight junctions [125]. It has been demonstrated that butyrate metabolism reduces local oxygen level which stabilizes HIF1α [125]. The basal HIF1α expression within the mucosa provides protection function, so existing a host-microbe crosstalk pathway wherein SCFAs may signal protective gut functions is important to maintain homeostasis to prevent the development of, e.g., metabolic disorders [125]. All these mechanisms and *WWOX/HIF1A* axis participation need further investigation to be potentially useful in therapy.

## 4. Materials and Methods

### 4.1. Cell Culture

1BR.3.N (ECACC 90020508) human skin fibroblasts (European Collection of Cell Cultures; catalog nr 90020508) were cultured in MEM (Gibco, Life Technologies, Singapore) medium containing 10% fetal bovine serum FBS (Thermo Fisher Scientific, Naarden, The Netherlands), 1% L-glutamine, 1% Non-essential aminoacids (NEAA) and 1% Penicillin, Neomycin and Streptomycin (Thermo Fisher Scientific, Naarden, The Netherlands). The cell line was maintained at 37 °C and 5% CO_2_ in a humidified atmosphere.

### 4.2. Stable Transduction

In order to receive a generation of *WWOX* downregulation cell line, cells were transfected by Human *WWOX* sgRNA CRISPR/Cas9 (pLenti-U6-sgRNA-SFFV-Cas9-2A-Puro) set and simultaneously we received control by transfecting by Scrambled sgRNA CRISPR/Cas9 Lentivirus (Applied Biological Materials Inc., Richmond, BC, Canada.). Additionally, the cell line was transfected with pLenti-GIII-CMV-GFP-2A-Puro lentivirus vector (Applied Biological Materials Inc., Richmond, BC, Canada), in two variants: a vector carrying human *WWOX* cDNA and a Puro-Blank Lentivirus as an experiment control (Applied Biological Materials Inc., Richmond, BC, Canada). Polybrene at a concentration of 8 μg/μL was added to the infection medium to enhanced the efficiency of lentiviral gene transduction. Both transductions were performed in a starving medium containing lentiviral particles at MOI = 2. In all vectors, a puromycin resistance gene was included and the selection was carried out with puromycin at a concentration of 1 μg/mL for four doses. Transfection efficiency was verified by RT-qPCR and western blot analysis.

### 4.3. Cell Culture Conditions

All variants of 1BR.3.N cells were seeded at a concentration of 3.0 × 10^4^ cells/cm^2^. During experiments all variants of the cell line were culture in four different conditions:

- normoxia (19% O_2_) and normoglycemia (5.5 mM glucose)
- normoxia (19% O_2_) and hyperglycemia (25 mM glucose)
- hypoxia (1% O_2_) and normoglycemia (5.5 mM glucose)
- hypoxia (1% O_2_) and hyperglycemia (25 mM glucose)

**Figure 12:**
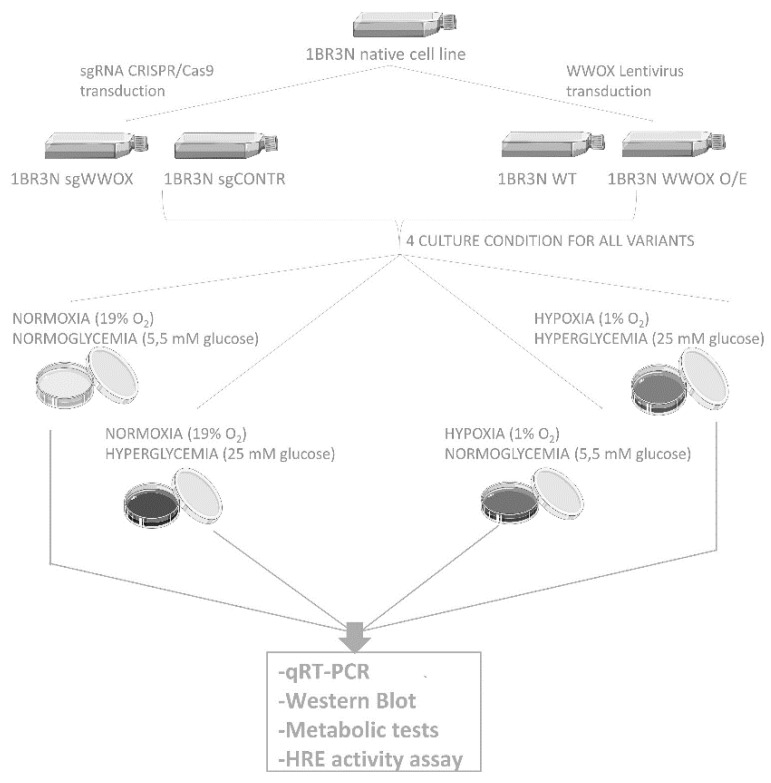
The scheme of experiment.

For 6 h or 48 h and then were examined. A summary of the experimental design was illustrated in Figure 12.

### 4.4. RT-qPCR

Total RNA was isolated from cells using TRIzol reagent (Invitrogen Life Technologies, Carlsbad, CA, USA) and then the cDNA synthesis was performed using ImProm RT-II™ reverse transcriptase (Promega Corporation, Madison, WI, USA) from 10 μg of total isolated RNA according to the manufacturer’s instructions. cDNA samples were diluted with nuclease-free water and 2 μL was added to the RT-qPCR reaction. Real-time polymerase chain reaction amplifications were conducted for WWOX, HIF1A, glucose transporters (*SLC2A1, SLC2A4*), glycolytic genes (*HK2, PFK1, ENO1, PKM2, LDHA, PDK1*), tricarboxylic acid (TCA) cycle (*PDHA, CS, ACLY*) genes using Light-Cycler 480 (F. Hoffmann-La Roche AG, Basel, Switzerland) with the SYBR Green dye I nucleic acid stain (Promega) according to the manufacturer’s instructions. All reactions were performed in duplicate. The relative level of expression were normalized to 3 reference housekeeping genes (H3F3A, RPLP0, RPS17) and the results were calculated by the Pfaffl method. Universal Human Reference RNA (Stratagene) was used as a calibrator.

### 4.5. Western Blot

After selection, variants of 1BR.3.N cell line were lysed in RIPA protein extraction buffer supplemented with protease inhibitor cocktail, Sodium orthovanadate and PMSF (Sigma-Aldrich, St. Louis, MO, USA) to confirm transduction results. After being lysed for 30 min on ice, samples were centrifuged at 14,000× g at 4 °C for 30 min. Supernatant was separated and the protein concentration was measured using the Bradford method (Bio-Rad Laboratories, Hercules, CA, USA). The proteins samples were separated on 10% SDS-polyacrylamide gel and then were transferred to a PVDF membrane (Sigma-Aldrich, St. Louis, MO, USA) for detection with primary antibodies for WWOX (Thermo Fisher Scientific, Naarden, the Netherlands, 1:100), HK2 (Genetex, Irvine, CA, USA; GTX111525, 1:10,000), PKM2 (ProteinTech, Manchester, United Kingdom; 15822-1-AP, 1:2000), LDHA (Genetex, Irvine, CA, USA; GTX101416, 1:3000), GLUT1 (ProteinTech, Manchester, United Kingdom; 21829-1-AP, 1:1000). Glyceraldehyde-3-phosphate dehydrogenase (GAPDH) (Santa Cruz Biotechnology Inc., Dallas, TX, USA.) at a dilution of 1:1000 or beta Actin (Genetex, Irvine, USA; GTX109639) at dilution of 1:10,000 were used as the reference proteins. After incubation with primary antibodies, the membranes were washed three times with TBST buffer (250 mM Tris-HCL pH 7.6, 1.5 M NaCl, 0.01% Tween) and incubated with secondary antibodies conjugated with alkaline phosphatase (Sigma-Aldrich, St. Louis, MO, USA) for 1 h. The Novex^®^ AP Chromogenic Substrate (Invitrogen Life Technologies, Carlsbad, CA, USA) was used to developed membranes. The optical density of bands was analyzed using the ImageJ software

### 4.6. Cytoplasmic and Nuclear Subfraction Isolation

After 6h culture in tested conditions the cytoplasmic and nuclear subfractions were isolated using a commercial kit NE-PER Nuclear and Cytoplasmic Extraction Reagents (Thermo Fisher Scientific, Naarden, the Netherlands) according to the manual. The concentration of the isolated protein was assessed by the Thermo Scientific Pierce BCA Protein Assay Kit. The clearance of the isolated subfraction was confirmed by western blot analyses with an anti-α-Tubulin antibody for the cytoplasmic subfraction and anti-Lamin A/C for the nuclear subfraction. The isolated proteins were stored at −80 °C for further analyses. Western blot was carried out as above for detection with primary antibodies for WWOX (Thermo Fisher Scientific, Naarden, The Netherlands, 1:100) and HIF1α (Genetex, Irvine, CA, USA, 1:2000) proteins. The optical density of bands was analyzed using the ImageJ software.

### 4.7. L-Lactate Assay

Lactate levels were determined with EnzyChrom Lactate Assay Kit (BioAssay Systems, Hayward, CA, USA) after 48 h cell culture in all tested conditions. Optical density (OD) was measured at 565 nm, for time “zero” and after 20 min incubation at room temperature. The intensity of the product color is proportionate to the lactate concentration in the sample.

### 4.8. Lactate Dehydrogenase

Lactate dehydrogenase activity was measured spectrophotometrically with QuantiChrom Lactate Dehydrogenase kit (BioAssay Systems, Hayward, CA, USA) after 48 h incubation in four different culture conditions. Optical density (OD) was measured at 565 nm and LDH activity was calculated according to the manufacturer’s instructions.

### 4.9. Hexokinase Activity

The HK activity was measured spectrophotometrically through NAD reduction in the glucose-6-phosphate dehydrogenase-coupled reaction by Hexokinase Colorimetric Assay Kit (BioVision, Milpitas, LA, USA). The absorbance was continuously recorded for 40 min at 450 nm. The activity was expressed in mU per mL of cells homogenate.

### 4.10. Pyruvate Dehydrogenase Activity

The assay is based on pyruvate dehydrogenase’s ability to convert pyruvate into acetyl-CoA and was determined by Pyruvate Dehydrogenase Activity Colorimetric Assay Kit (BioVision, Milpitas, LA, USA). The assay tested pyruvate dehydrogenase, which converting pyruvate into an intermediate, which reduces the developer to a colored product, which was measured at 450 nm. Pyruvate dehydrogenase activity was calculated based on NADH Standard Curve and expressed in mU/mL.

### 4.11. Citrate Synthase Activity

Citrate synthase activity were found out with Citrate Synthase Activity Colorimetric Assay Kit (BioVision, Milpitas, LA, USA) after 48 h cell culture in all tested conditions. Optical density (OD) was measured at 412 nm in kinetic mode for 40 min in room temperature and CS activity was calculated according to the manufacturer’s instructions in U/mL.

### 4.12. Glucose (2-DG) Uptake

Glucose uptake was detected by a glucose analog 2-deoxyglucose in 1BR.3.N normal fibroblasts. Briefly, all cells’ variants were incubated in free FBS for 48 h in all tested conditions. The medium was displaced with KRPH buffer pH 7.4 containing 2% BSA, then added 2-DG (final concentration of 1 mM) and stimulated or not with 1 µM insulin. Optical density (OD) was measured at 412 nm at 37 °C every 5 min and glucose activity was calculated according to the manufacturer’s instructions in µM.

### 4.13. Luciferase Reporter Assay

All obtained transduced variants of 1BR.3.N cells were cotransfected using FUGENE HD transfection reagent (Promega Corporation, Madison, WI, USA) with 1 μg of pGL4.42 [luc2P/HRE/Hygro] vector (Promega Corporation, Madison, WI, USA) together with 0.1 μg of pGL4.75 Renilla luciferase reporter vector (Promega Corporation, Madison, WI, USA), used as a normalization control. Transfection was performed according to the manufacturer’s instruction. The cells were afterwards cultured at standard condition (5% CO_2_; 37 °C) for 24 h. Then, the transfection’s medium were removed and 75 µL of the medium was added: to the first one 5mM glucose concentration and 25 mM to the other. One of the plates was then incubated for 6h under hypoxia conditions (1% O_2_) and the next under normoxic conditions (19% O_2_). After incubation, the luciferase activity in each group was measured using the Dual-Glo Luciferase Assay System (Promega Corporation, Madison, WI, USA) by the VICTOR Multilabel plate reader (PerkinElmer, Waltham, MA, USA).

### 4.14. Immunocytochemistry

Cells were grown on glass coverslips at a seeding density of 2 × 104 cells/cm^2^. After culture in all tested conditions, the cells were fixed with ice-cold 4% PFA prepared in PBS for 10 min. After washing in PBS (3 times, each for 5 min), non-specific antibody binding was blocked with blocking buffer (2% BSA, 5% donkey serum, and 0.1% Triton-X100). The slides with cells were then incubated overnight at 4 °C with the primary antibodies in solution (dilution 1:100 for HIF1α (Genetex, Irvine, USA, GTX127309) and 1:500 for WWOX (Thermo Fisher Scientific, Naarden, The Netherlands, PA5-29701)) in 5% donkey serum. After double washing with PBS, the cells were incubated 1h at 37 °C with secondary antibodies (donkey, Alexa Fluor 594, catalog number: A21207), diluted 1:1000. The nuclei were counterstained with ProLong™ Gold reagent with DAPI (Thermo Fisher Scientific, Naarden, The Netherlands), the cells were then imaged at 40× magnification, using an OPTA-TECH MN 800 fluorescent microscope. Quantification of WWOX and HIF1α fluorescence was performed using the ImageJ integrated density measurement. Cells were casually chosen along with areas representing background fluorescence. The fluorescence signal was calculated using the formula for Total Corrected Cell Fluorescence (TCCF) [127]:

Integrated Density–(area of selection × mean background fluorescence)

Results were compared in all types of tested conditions using one-way ANOVA with Tukey’s post-hoc test on Graphpad Prism. Data are represented as mean ± SEM.

### 4.15. Statistics Analysis

In vitro data are reported as mean +/− SEM. Differences in gene and protein expression, enzymatic activity were analyzed by one-way ANOVA with repeated measures, followed by the Tukey’s post-hoc test. Two group comparisons were made using a 2-tailed unpaired Student’s t-test for probes with for equal variances and 2-tailed unpaired Student’s t-test with Welch’s correction for probes with heterogeneity of variance (Transfection confirmation, HRE luminescence test). Statistical significance was set at levels of *p* < 0.05, *p* < 0.01 and *p* < 0.001.

## 5. Conclusions

Diabetes is a very complicated disease whose pathology includes a complex combination of genetic, metabolic, and environmental factors that cooperate with each other contributing to its onset. Faced with a global epidemic of type 2 diabetes, it is demanding to improve knowledge of T2D pathogenesis. Our previous studies with GDM patients indicated a significant role of WWOX in the coordination of glucose metabolism through the regulation of HIF1A. In this research, we explored the molecular basis on cellular level of this event. We proposed that *WWOX* downregulation is a key early component of diabetic pathophysiology. The Warburg effect, as a cellular energetic alteration resulting from decreased *WWOX* expression, probably is an early and essential factor of a metabolic process that mediates the hyperglycemia occurrence. The obtained results clearly show that decreased expression of *WWOX* is highly likely to be associated with a predisposition to the development of metabolic disorders such as gestational diabetes and type II diabetes. This is one of the first reports of the participation of the *WWOX/HIF1A* axis and the Warburg effect in the pathogenesis of diabetes, therefore further research is definitely needed, which could indicate the potential clinical use of the obtained information. However, strategies to normalize glucose metabolism by targeting *WWOX* may have promise as therapies in the future.

## Data Availability

The obtained data supporting the conclusions of this article are included within the article and its Supplementary files.

## Supplementary Materials

The following supporting information can be downloaded at: https://www.mdpi.com/article/10.3390/ijms23063326/s1.
