# Supplementary material for: The WWOX/HIF1A Axis Downregulation Alters Glucose Metabolism and Predispose to Metabolic Disorders
Source: Int J Mol Sci. 2022 Mar 19;23(6):3326. doi: 10.3390/ijms23063326 (PMC8955937; doi:10.3390/ijms23063326)
Supplement: Supplementary file 1 [file ijms-23-03326-s001.zip › Supplementary figures captions.pdf]

Supplementary figures captions.

Figure S1: Luciferase reporter assay of hypoxia response element and glucose uptake in WWOX overexpression fibroblasts. (a) Obtained variants of 1BR.3.N cell line (1BR.3.N WT and 1BR.3.N WWOX OE) was transfected with a luciferase reporter gene under the regulation of Hypoxia-Responsive Elements (HRE) and subjected to all tested conditions for 6 hours. Extracts were analyzed for luciferase activity. The values are mean ± SEM. *, p<0.05; **, p<0.01; ***, p<0.001. (b) Glucose uptake in WWOX OE and WT variants of 1BR.3.N cell line incubated all tested conditions (Normoxia Normoglycemia, Normoxia Hyperglycemia, Hypoxia Normoglycemia, Hypoxia Hyperglycemia) with or without insulin for 48 hours. The values are mean ± SEM. *, p<0.05; **, p<0.01; ***, p<0.001.

Figure S2: Relative expression fold change of glycolysis-associated genes detected by RT-qPCR in WWOX-overexpressing fibroblasts after 48h incubation in four tested condition. Bar graphs showing the relative mRNA expression (the ratio of the target gene relative to the reference genes RPS17, RPLP0, H3F3A) of WWOX (a), HIF1A (b), the WWOX/HIF1A ratio (c) and all WWOX/HIF-related genes including those involved in glucose transport (SLC2A1 (d), SLC2A4 (e)), glycolytic pathway (HK2 (f), ENO1 (g), PFK (h), PKM2 (i), LDHA (j)), PDK (k), PDHA (l), CS (m) and ACLY (n). Results of ANOVA with a post-hoc Tukey-Test are shown as mean ± SEM. *, p<0.05; **, p<0.01; ***, p<0.001.

Figure S3: The activity of glycolysis enzymes and lactate concentration in WWOX-overexpressing fibroblasts after 48h incubation in four tested condition. Bar graphs showing the enzyme activity of Hexokinase (a), Pyruvate Dehydrogenase (b), Lactate Dehydrogenase (c) Citrate Synthase (e) and Lactate concentration (d). Results of ANOVA with a post-hoc Tukey-Test are shown as mean ± SEM. *, p<0.05; **, p<0.01; ***, p<0.001.

Figure S4: WWOX and HIF1α western blot analysis of cytoplasmic (a) and nuclear (b) fractions of WT and WWOX OE 1BR.3.N cells after 48h incubation in four tested condition. Results of ANOVA with a post-hoc Tukey-Test are shown as mean ± SEM. *, p<0.05; **, p<0.01; ***, p<0.001; NN - normoxia normoglycemia, NH – normoxia hyperglycemia, HN – hypoxia normoglycemia, HH – hypoxia hyperglycemia.

Figure S5: The western blot analysis of selected proteins (HK2, PKM2, LDHA, GLUT1) of 1BR.3.N WWOX OE and 1BR.3.N WT cells after 48h incubation in four tested condition (a). Bar graphs showing the relative level OD density of HK2 (b), PKM2 (c), LDHA (d), GLUT1 (e) to ACTIN. Results of ANOVA with a post-hoc Tukey-Test are shown as mean ± SEM. *, p<0.05; **, p<0.01; ***, p<0.001. NN - normoxia normoglycemia, NH – normoxia hyperglycemia, HN – hypoxia normoglycemia, HH – hypoxia hyperglycemia.

Figure S6: Immunocytochemistry analysis of WWOX and HIF1α in 1BR.3.N WWOX OE and WT cells after 6h incubation in normoxia (a) and hypoxia (b). Calculation of Total Corrected Cell Fluorescence (TCCF) (c). Results of ANOVA with a post-hoc Tukey-Test are shown as mean ± SEM. *, p<0.05; **, p<0.01; ***, p<0.001.
