# Supplementary figures and images for: The WWOX/HIF1A Axis Downregulation Alters Glucose Metabolism and Predispose to Metabolic Disorders
Source: Int J Mol Sci. 2022 Mar 19;23(6):3326. doi: 10.3390/ijms23063326 (PMC8955937; doi:10.3390/ijms23063326)

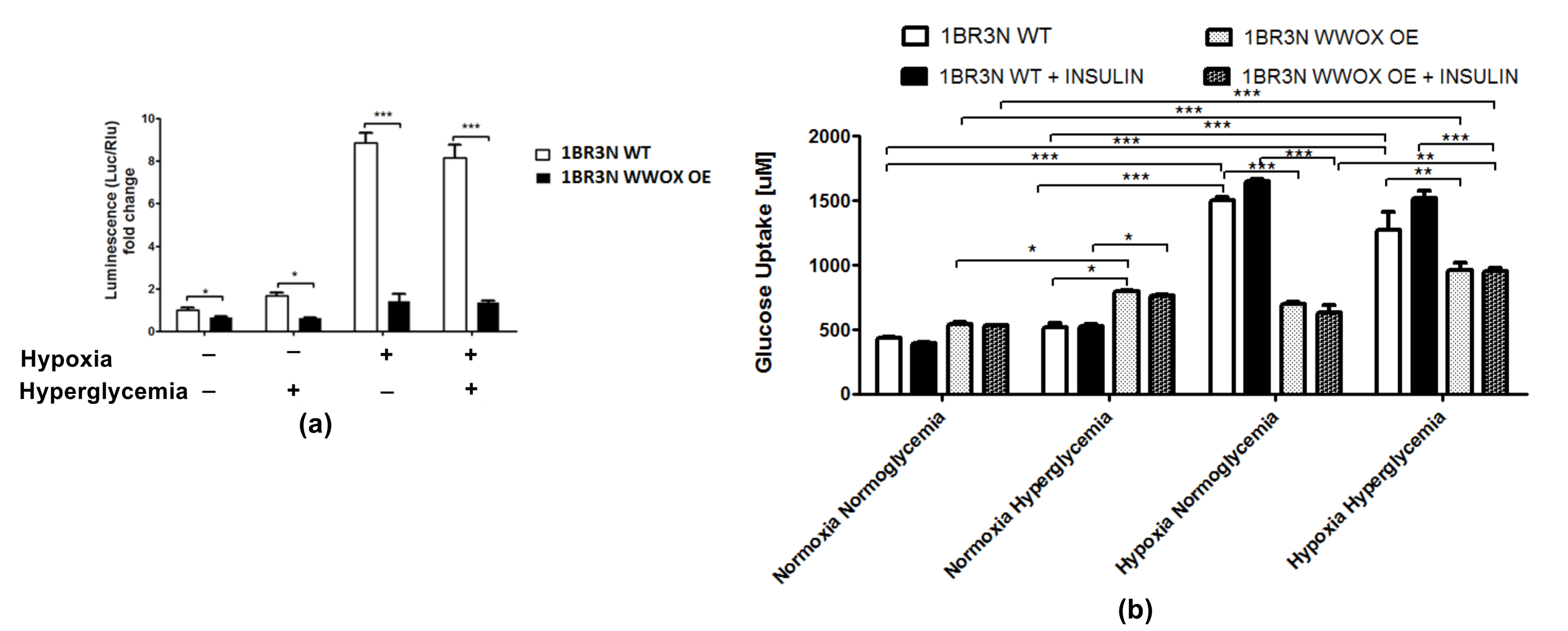

Supplement: Supplementary file 1 [file ijms-23-03326-s001.zip › Figure S1.tif]

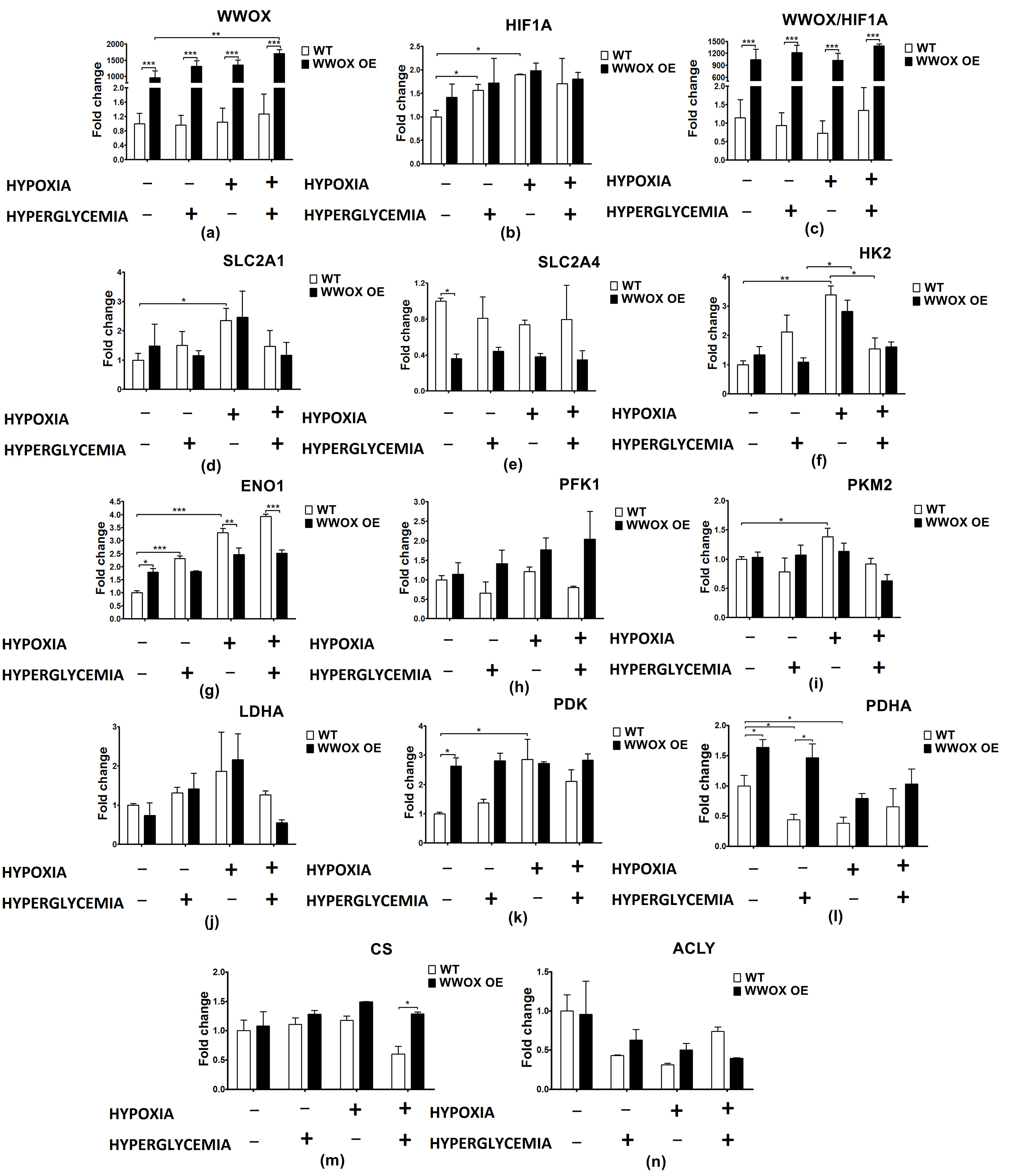

Supplement: Supplementary file 1 [file ijms-23-03326-s001.zip › Figure S2.tif]

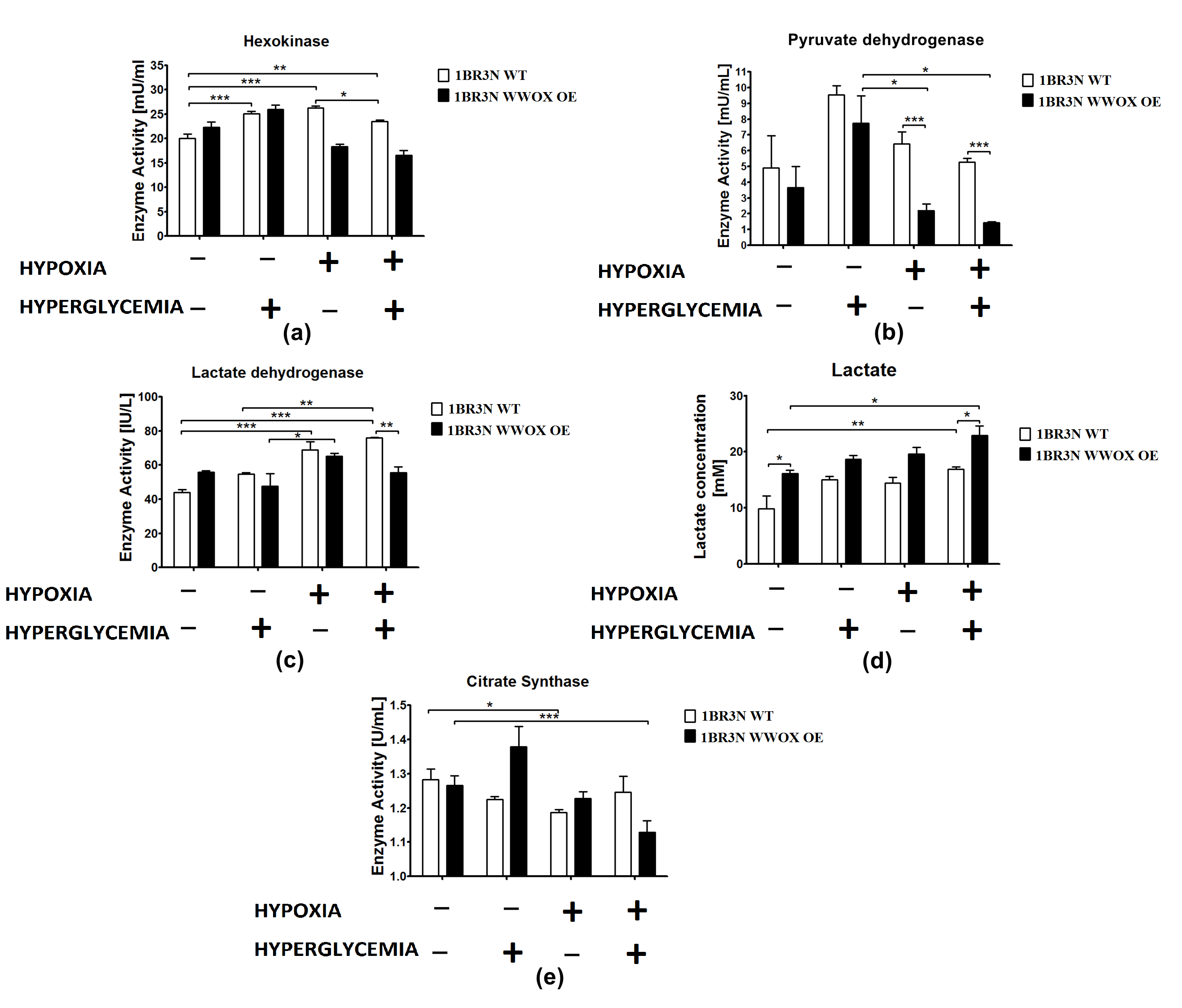

Supplement: Supplementary file 1 [file ijms-23-03326-s001.zip › Figure S3.tif]

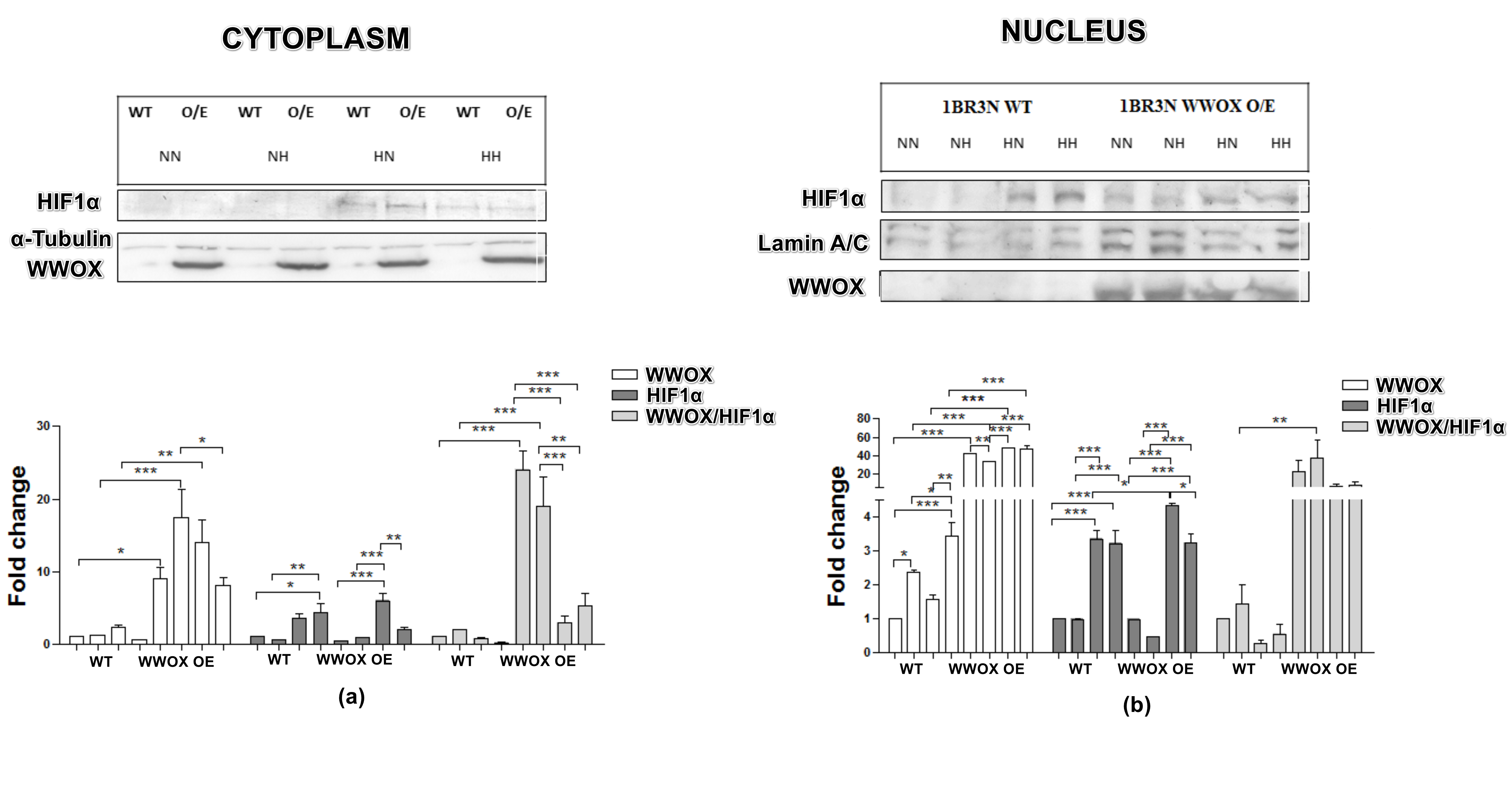

Supplement: Supplementary file 1 [file ijms-23-03326-s001.zip › Figure S4.tif]

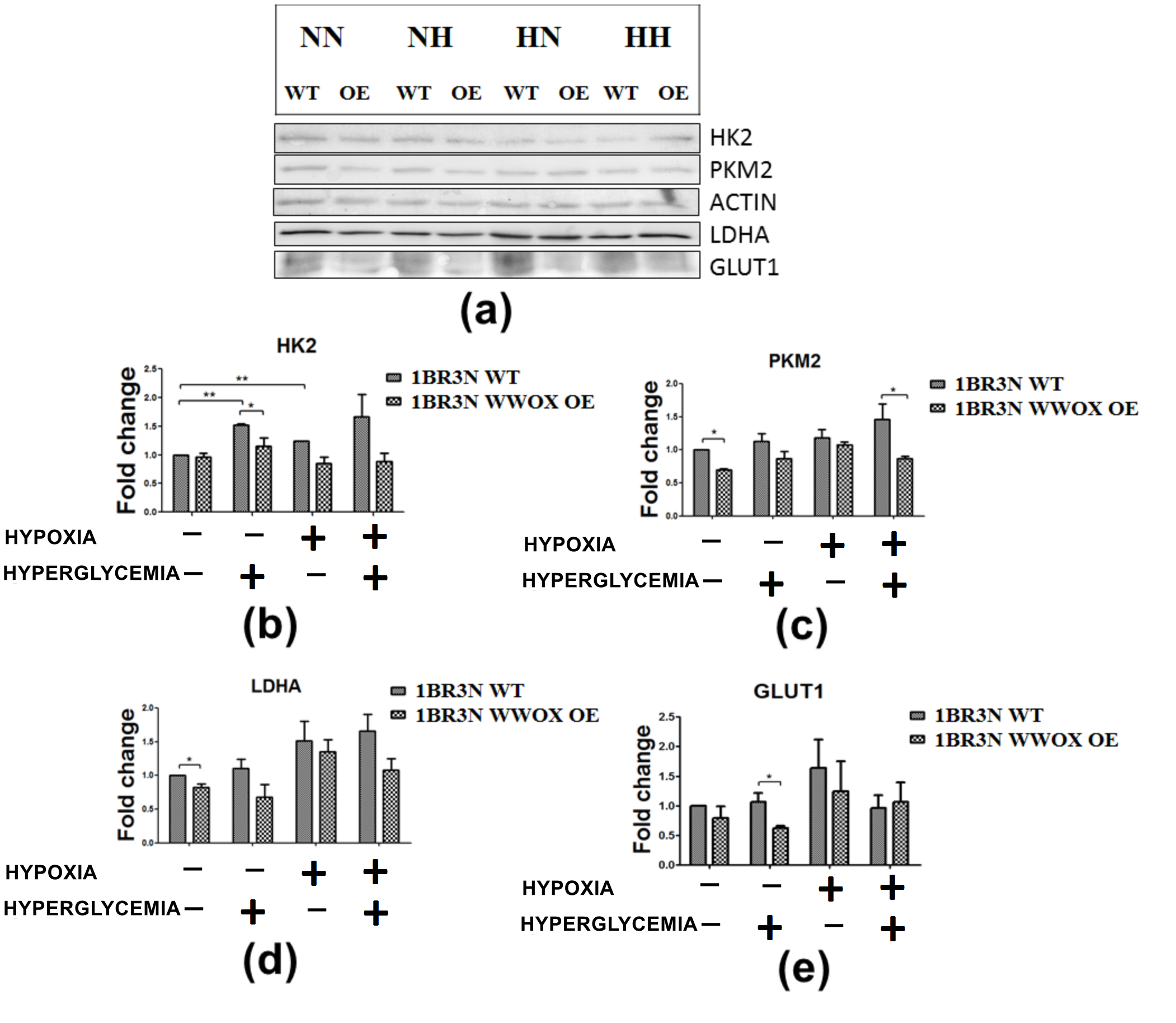

Supplement: Supplementary file 1 [file ijms-23-03326-s001.zip › Figure S5.tif]

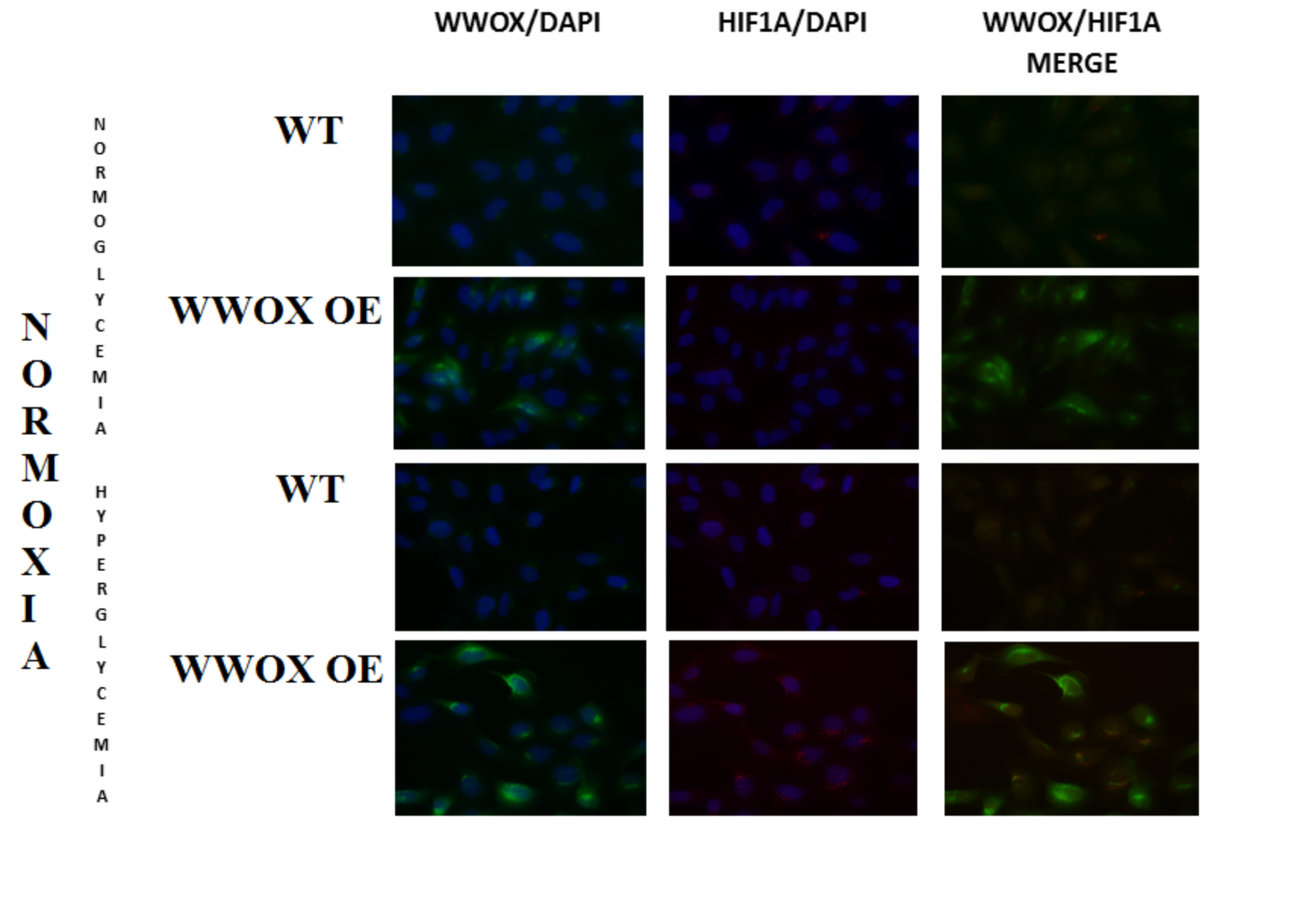

Supplement: Supplementary file 1 [file ijms-23-03326-s001.zip › Figure S6A.tif]

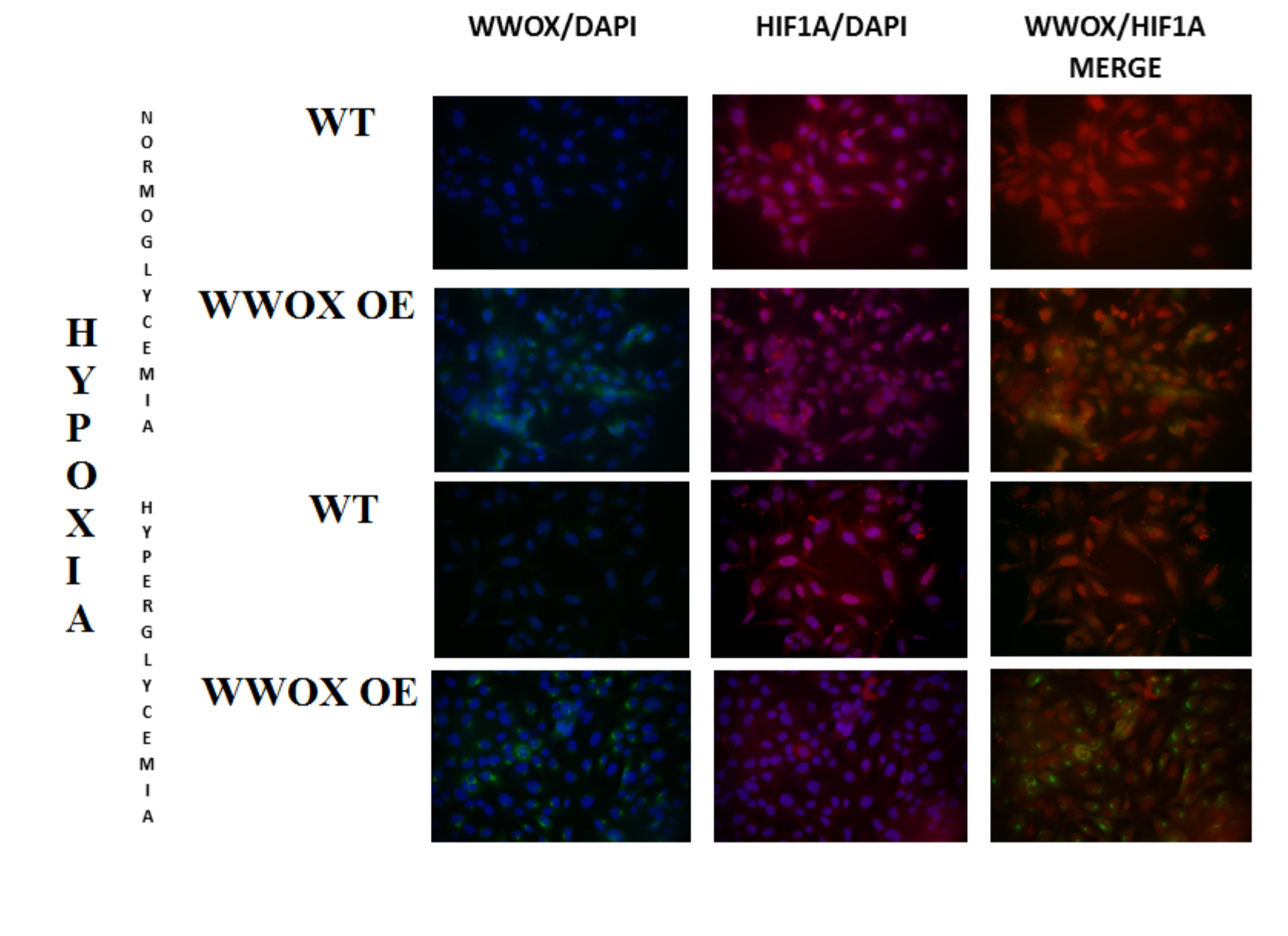

Supplement: Supplementary file 1 [file ijms-23-03326-s001.zip › Figure S6B.tif]

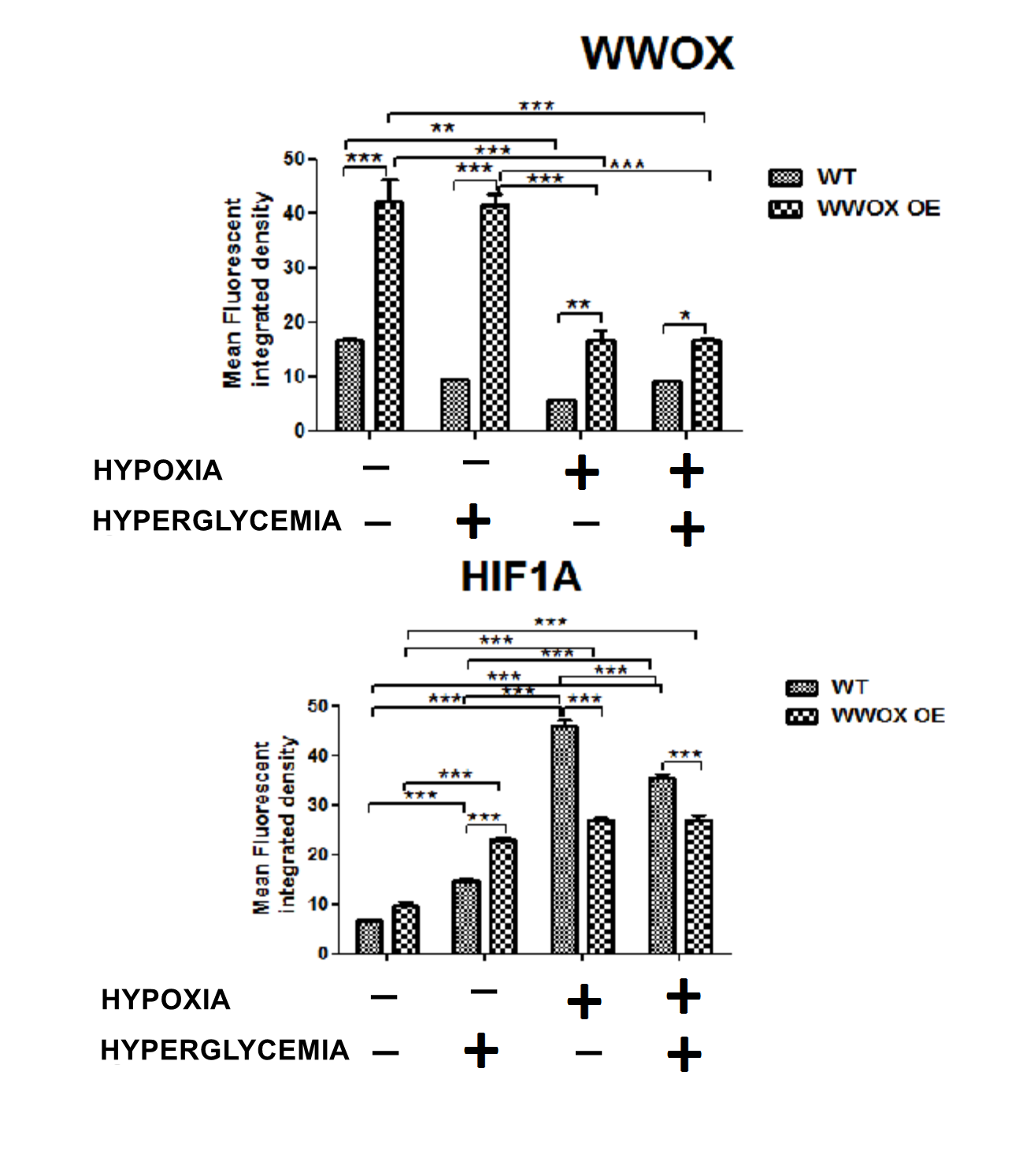

Supplement: Supplementary file 1 [file ijms-23-03326-s001.zip › FigureS6C.tif]
